# Phylogeny, Evolution, and Transmission Dynamics of Canine and Feline Coronaviruses: A Retro-Prospective Study

**DOI:** 10.3389/fmicb.2022.850516

**Published:** 2022-04-26

**Authors:** Hu Yang, Qianling Peng, Yifei Lang, SenYan Du, SanJie Cao, Rui Wu, Qin Zhao, Xiaobo Huang, Yiping Wen, Juchun Lin, Shan Zhao, Qigui Yan

**Affiliations:** ^1^Swine Disease Research Center, College of Veterinary Medicine, Sichuan Agricultural University, Chengdu, China; ^2^Key Laboratory of Animal Disease and Human Health of Sichuan Province, Sichuan Agricultural University, Chengdu, China; ^3^Department of Basic Veterinary Medicine, College of Veterinary Medicine, Sichuan Agricultural University, Chengdu, China

**Keywords:** canine coronavirus, feline coronavirus, DAPC, recombination, phylogeny, phylodynamics, BEAST

## Abstract

Canine coronavirus (CCoV) and feline coronavirus (FCoV) are endemic in companion animals. Due to their high mutation rates and tendencies of genome recombination, they pose potential threats to public health. The molecular characteristics and genetic variation of both CCoV and FCoV have been thoroughly studied, but their origin and evolutionary dynamics still require further assessment. In the present study, we applied a comprehensive approach and analyzed the S, M, and N genes of different CCoV/FCoV isolates. Discriminant analysis of principal components (DAPC) and phylogenetic analysis showed that the FCoV sequences from Chinese isolates were closely related to the FCoV clusters in Netherlands, while recombination analysis indicated that of S N-terminal domain (NTD) was the most susceptible region of mutation, and recombination of this region is an important cause of the emergence of new lineages. Natural selection showed that CCoV and FCoV subgenotypes were in selection constraints, and CCoV-IIb was in strong positive selection. Phylodynamics showed that the mean evolution rate of S1 genes of CCoV and FCoV was 1.281 × 10^–3^ and 1.244 × 10^–3^ subs/site/year, respectively, and the tMRCA of CCoV and FCoV was about 1901 and 1822, respectively. Taken together, our study centered on tracing the origin of CCoV/FCoV and provided ample insights into the phylogeny and evolution of canine and feline coronaviruses.

## Introduction

Coronaviruses are notorious pathogens that can infect a wide range of vertebrate hosts. Characterized by their propensity to cross species barriers, they pose a prolonged threat to both public health and the animal breeding industry. Indeed, the twenty-first century had already witnessed the emergence of three novel coronaviruses, namely, SARS-CoV, MERS-CoV, and, most recently, SARS-CoV-2 that spread throughout the globe and claimed over five million lives. Coronavirus constitutes the subfamily *Orthocoronavirinae*, which further belongs to the family *Coronaviridae* that lies within the suborder *Coronavirineae*, order *Nidovirales*. Based on the genetic and serological characteristics of coronaviruses, the subfamily *Orthocoronavirinae* is classified into four genera: *Alphacoronavirus* (αCoV), *Betacoronavirus* (βCoV), *Gammacoronavirus* (γCoV), and *Deltacoronavirus* (δCoV) (Masters, 2006; Zhang et al., 2021).

Canine and feline coronaviruses are widespread in both domestic and wild animals (Arshad et al., 2004; Stavisky et al., 2010). Therefore, investigations on canine coronavirus (CCoV) and feline coronavirus (FCoV) have more potential public health significance. Both FCoV and CCoV are members of the *Alphacoronavirus* genus, together with transmissible gastroenteritis virus (TGEV) and porcine epidemic diarrhea virus (PEDV). FCoV and CCoV are enveloped viruses that have positive-stranded RNA genomes which are 28.4–30 kb in size (Masters, 2006). The genome contains 11 open reading frames (ORFs) in the order of 5′UTR-ORF1a-ORF1b-S-ORF3a/b/c-E-M-N-ORF7a/b-3′UTR in genome structure (Le Poder, 2011). They encode replicase-associated protein, spike protein, accessory proteins 3a, 3b, and 3c, envelope protein, membrane protein, nucleocapsid protein, and accessory proteins 7a and 7b (Le Poder, 2011). The four major structural proteins (S, E, M, and N) are associated with viral components, invasive infection, and immune activation and are also major targets in many research articles and vaccine applications (Shchetinina et al., 1995). FCoV is divided into two biotypes: the non-pathogenic feline enteric coronavirus (FECV) and the highly virulent feline infectious peritonitis virus (FIPV) (Jaimes et al., 2020). CCoV only has one biotype, but it differs in pathogenicity and is henceforth divided into pantropic variant virulent strains and common attenuated strains, causing varying levels of gastroenteritis symptoms in dogs of different ages (Decaro et al., 2007, 2013).

Feline coronavirus can be classified as FCoV type I (FCoV-I) and FCoV type II (FCoV-II), based on genetic divergence in the S gene, and the same holds for CCoV (CCoV-I and CCoV-II) (Herrewegh et al., 1998; Pratelli et al., 2003). CCoV-II can be divided into CCoV-IIa, CCoV-IIb, and CCoV-II variant (CCoV-IIv), though it was not officially recognized (Regan et al., 2012). FCoV-I and CCoV-IIa are the most classical strains, while the FCoV-II, CCoV-I, CCoV-IIb, and CCoV-IIv are novel recombinant strains due to S gene recombination (Le Poder, 2011). It is commonly known that S is not only a criterion for inter-lineage classification, but also an important epitope and evolutionary marker for virus invasion and immune evasion, particularly in driving cross-species transmission and host adaption (Belouzard et al., 2012).

In recent years, many researchers made use of genetic information to decipher the origin of different viruses, such as phylogenetic and Bayesian phylodynamic methods to reveal the transmission dynamics and evolutionary history of avian influenza virus H1N1 (Su et al., 2015) and H5N6 (Zhang J. et al., 2020), brassica pathogen turnip mosaic potyvirus (TuMV) (Kawakubo et al., 2021), potato virus Y (PVY) (Gao et al., 2020), and porcine deltacoronavirus (PDCoV) (He et al., 2020). However, similar studies on CCoV and FCoV are still lacking.

To further understand the molecular epidemiology and evolutionary history of CCoV and FCoV, we collected fecal and ascites samples from different areas in Sichuan province, China. S1, M, and N genes were identified to examine population genetics and phylogeny. Our data revealed the relationship between population genetic principal components, gene recombination, and evolution rate and provided insights into potential amino acid sites that could potentially drive virus evolution and host adaption of FCoV and CCoV in China.

## Materials and Methods

### Sample Collection, Sequencing, and Primary Analysis

Fecal samples were obtained from diseased and healthy cats and dogs in Sichuan province. Ascites samples were obtained from cats developing peritonitis in Sichuan province. Viral RNA genomes were extracted using Sangon Viral RNA Kit (Sangon, Shanghai, China) and stored at −80°C. The cDNA was synthesized using the Vazyme HiScript III RT SuperMix Kit (Vazyme, Nanjing, China) or the RT Easy™ I Master Premix Kit (Foregene, Chengdu, China). FCoV and CCoV were detected using the previously reported primers (Herrewegh et al., 1995; Naylor et al., 2001). The S1 gene, N gene, and M gene of the positive samples were amplified with the PrimeSTAR ^®^ Max DNA Polymerase (Takara, Japan). Purified RT-PCR products were cloned into the pMD-19T vector. The positive recombinant plasmids were sent for sequencing (Sangon, Shanghai, China). Multiple sequence alignment was performed using MACSE (Ranwez et al., 2011) in PhyloSuite (version 1.2.2) (Zhang D. et al., 2020) and then manually adjusted in MEGAX (Kumar et al., 2018).

A nucleotide identity matrix was generated by Bio-Aider (Zhou et al., 2020). Nucleotide diversity (π) and haplotype diversity (Hd) of FCoV were calculated using DNAsp5 (Librado and Rozas, 2009). F_ST_, a population genetic differentiation value, was calculated using Arlequin 3.5 (Excoffier and Lischer, 2010). K_ST_ and Snn values were calculated using DNAsp5 (Librado and Rozas, 2009). The hypothesis of deviation from zero population differentiation was implemented by 1,000 permutations of the initial data.

### Discriminant Analysis of Principal Components

The discriminant analysis of principal components (DAPC) for FCoV was performed with the R package and adegenet library by geographic location (Jombart et al., 2010). Due to insufficient CCoV samples in different geographic regions, only FCoV DAPC analysis was completed. The dataset was first imported and transformed based on PCA. K-means algorithm was performed for clustering, and LD analysis was performed to evaluate the clustering condition under different *K* values.

### Recombination Analysis

Gene recombination analysis was performed using RDP5 (Martin et al., 2021) for different S1, N, and M genes. According to previous reports, TGEV is also involved in the recombination; hence, the corresponding gene sequences of TGEV were added. We used seven methods such as RDP (Martin and Rybicki, 2000), 3Seq (Lam et al., 2018), GENECONV (Sawyer, 1999), Chimera (Posada and Crandall, 2001), SiScan (Gibbs et al., 2000), MaxChi (Smith, 1992), and BootScan (Salminen et al., 1995) in RDP5. Credible recombination events could be detected by at least three of the seven methods with a *P*-value cutoff of 10^–6^. Simplot was applied to further show the breakpoint position of the recombination event.

### Phylogenetic Analysis

The maximum likelihood phylogeny for S1, M, and N genes was inferred using IQTREE version 2.1.3 (Nguyen et al., 2015) in PhyloSuite (version 1.2.2) (Zhang D. et al., 2020) with SeACoV reference sequence as outgroup. The substitution model calculated by ModelFinder (Kalyaanamoorthy et al., 2017) in PhyloSuite (version 1.2.2) (Zhang D. et al., 2020) was GTR + F + R10 for S1 gene, GTR + F + R5 for N gene, and GTR + F + R6 for M gene. All of the maximum likelihood trees were with 10,000 ultrafast bootstrap replicates to estimate branch support. Finally, the ML trees were visualized and annotated in iTOL v6^1^.

### Natural Selection Analysis

To reveal the natural selection between the different lineages of FCoV and CCoV, the branch model was performed in EasyCodeML (version 1.4) (Gao et al., 2019) by a non-temporal ML tree and a PML format non-recombinant sequence file. In addition, to find the site that was positively selected on the S1 gene, we implemented a site model for each subgenotype sequence set. Only when the probability of identified positive sites is greater than 0.95, it could be credible.

### Phylodynamic Analysis

We performed the phylodynamic analysis using the S1 gene of FCoV-I and CCoV-IIa. Firstly, we used TempEst (version 1.5.3) to preliminarily assess a temporal signal of S1 gene dataset and then remove some sequences with an abnormal temporal signal. Finally, we used Bayesian evaluation of temporal signal (BETS) (Duchene et al., 2020) method to evaluate the final time structure. The generalized stepping-stone sampling method was performed in BEAST (version 1.10.4) to obtain the marginal likelihood estimation of heterochronous model (Mhet) and isochronous model (Miso) and infer Bayes factor (BF). If log BF > 5, it meant that the dataset had an excellent temporal structure.

Path-sampling and stepping-stone sampling methods are used to calculate the best molecular clock model and tree prior model (Suchard et al., 2018). The result showed that the strict clock model with the Bayesian Skygrid coalescent model (Gill et al., 2013) is the best model for FCoV S1. Also, the uncorrelated lognormal relaxed clock model with the Bayesian skyline coalescent model (Minin et al., 2008) is the best model for CCoV S1. BEAST runs were performed using four independent chains with a chain length of 1 × 10^8^ for FCoV and one chain with a chain length of 5 × 10^7^ for CCoV with GTR + F + I + G4 substitution model. After data convergence (ESS > 200) in Tracer (version 1.7.1), the log and tree file were mixed by LogCombiner (version 1.10.4) with a burn-in period of 10% total chain length and all trees. The MCC tree was annotated by TreeAnnotator (version 1.10.4) and visualized by Figtree (version 1.4.4). In addition, we used Skygrid analysis and Bayesian skyline analysis by Tracer (version 1.7.1) to reconstruct the effective FCoV and CCoV population history over time, respectively.

## Results

### Sequencing and Analysis

To allow better bioinformatics analysis, 28 S1 genes, 34 N genes, and 34 M genes (coding-complete sequences) were obtained from FCoV-positive samples, while 10 S1 genes, 12 N genes, and 12 M genes were obtained from CCoV-positive samples (Supplementary Table 1). The nucleotide sequences of the entire genome, S gene, M gene, and N gene of selected FCoV and CCoV strains were retrieved from GenBank (Supplementary Table 1).

The average nucleotide identity of FCoV S1, N, and M genes from Sichuan, China, was 79.71% (41.6–99.96%), 92.9% (90.04–99.91%), and 88.67% (79.82–99.87%) (Supplementary Tables 2–4), while that of CCoV was 81.18% (38.29–98.64%), 91.13% (77.11–99.91%), and 91.61% (80.88–99.49%), respectively (Supplementary Tables 5–7). In addition, the average nucleotide identity between FCoV and CCoV was 48.52% (39.66–99.05%), 80.59% (76.85–94.8%), and 80.1% (79.25–99.75%), respectively. We observed that the S1 gene of either FCoV or CCoV showed very low similarity (about 40% at the lowest), even within one species. Those findings suggest that there might be a genetic relationship between them or a gene exchange and co-evolution.

### Population Genetics and Genetic Principal Component Discrimination Analysis

We identified 22 haplotypes among the FCoV S1 genes in our study, with a haplotype diversity of 0.958 and a nucleotide diversity of 0.171 (Supplementary Table 8). The nucleotide diversity of FCoV S1 in Sichuan was lower than that of other isolates in China, Japan, and the United States, but higher than that of isolates in the United Kingdom and Netherlands (Supplementary Table 8). A total of 25 and 26 haplotypes were identified among the N and M genes in our study with a respective haplotype diversity of 0.975 and 0.979 and a nucleotide diversity of 0.063 and 0.108, respectively (Supplementary Table 8). By contrast, the nucleotide diversity of FCoV N and M genes in Sichuan was higher than that of other isolates in China. K_ST_, S_nn_, and F_ST_, all three population genetic differentiation values, were statistically significant (*p* < 0.05) (Supplementary Table 9). Compared with other strains in China, FCoV N genes in Sichuan were highly differentiated, followed by S genes (Supplementary Table 9). In general, Sichuan strains are rigorously differentiated in comparison with the strains in the United States and the United Kingdom, but were less differentiated than the Dutch strains.

Discriminant analysis of principal components showed that the optimal cluster *K* value of S1, N, and M genes was seven, four, and six, respectively (Supplementary Figures 1–3). It was obvious for all viral genes that Sichuan FCoV groups shared many genetic components with the Dutch FCoV groups, but only a few with the Japanese and American FCoV groups in terms of S, M, or N genes (Supplementary Figures 1–3). The DAPC scatter plot also indicated that Sichuan FCoV groups and other FCoV populations in China were genetically related to populations in Netherlands, Japan, and the United States (Supplementary Figure 4).

### FCoV and CCoV Have Extensive Genetic Recombination in S1 Gene

**FIGURE 1:**
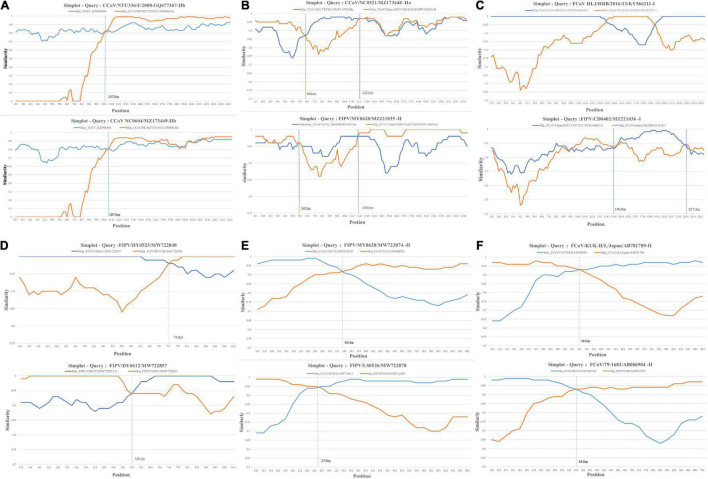
Simplot recombination analysis of S1, N, and M genes in our samples. Each point is the percent genetic similarity within a 200-nt-wide sliding window centered on the position plotted with a step size of 20 nt. The blue line indicates the percentage of similarity between the secondary parent and the recombination sequence, and the red line shows the percentage of similarity between the primary parent and the recombination sequence. **(A–C)** Recombination events that occur within the S1 gene. **(D)** Recombination events that occur within the N gene. **(E,F)** Recombination events that occur within the M gene.

We found multiple reorganization events, including those reported in previous studies and newly discovered. A total of 12 S1, 4 N, and 8 M gene recombination events were detected, respectively, by RDP5 (Supplementary Table 10). CCoV NC0604 had the same recombination pattern as the previously reported CCoV-IIb (Figure 1A). This was the first time that a recombination event had been reported in mainland China. The C-terminal domain of CCoV NC0604 S1 gene exhibited high similarity with CCoV-IIa, while the N-terminal domain exhibited high similarity with TGEV (Figure 1A). We found a putative recombination receptor-binding sequence “TTACTACAG” in CCoV NC0604 strain, which shared high similarity to the putative donor-binding sequence “TTACGCAA” (Decaro et al., 2009). In our study, recombination events also occurred between CCoV NC0521 and FIPV MY0628 in the N-terminal domain of the S1 gene (Figure 1B). This was similar to the recombination pattern of CCoV CB/05, because of their roughly same breakpoints (Supplementary Table 10). We also found that the recombination in the C-terminal domain, such as FIPV CD0402, was similar to CCoV HLJ/HRB/2016/13 (Figure 1C). Obviously, these recombination events did not occur among inter-lineages. In our study, we detected two N gene recombination strains (FIPV DY0523 and FIPV DY0612) through RDP5 (Figure 1D). The recombination breakpoint of FIPV DY0523 was 741 bp and, which was similar to FCoV UU30 (HQ392472). The recombination breakpoint of FIPV DY0612 was 581 bp, which was similar to PC/M477/06 strain (GU017103) (Figure 1D). It was obvious that the N gene recombination events occurred more often in the C-terminal domain. Similarly, we found that the M genes of FIPV LS0526 and FIPV MY0628 had recombination events at 271 and 341 bp, respectively, which were similar to other strains (FCoV KUK-H/L and FCoV 79-1683) (Figures 1E,F). These popular recombination events could be related to virus assembly, invasion, and pathogenicity.

### Analysis of Evolutionary Selection Inter-Lineage and Intra-Lineage

**FIGURE 2:**
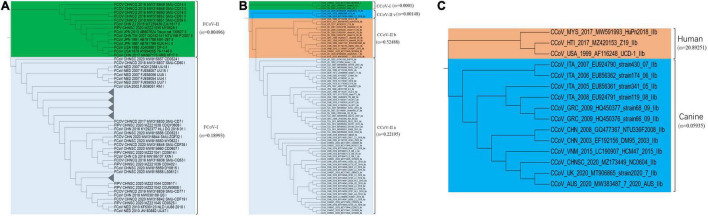
Natural selection analysis of inter-lineage. ω value means the ratio of non-synonymous to synonymous substitution rates (dN/dS). Using the branch model to calculate the selection constraints of different clades in EasyCodeML (version 1.4). **(A)** Comparison of selection constraints between FCoV-I and FCoV-II. **(B)** Comparison of selection constraints between CCoV-I, CCoV-IIv, CCoV-IIb, and CCoV-IIa. **(C)** Comparison of selection constraints between human-derived CCoV and canine-derived CCoV.

Natural selection pressure of inter-lineage was calculated by a branch model in EasyCodeML with FCoV-I ω = 0.18993 (Figure 2A), FCoV-II ω = 0.00496 (Figure 2A), CCoV-I ω = 0.0001 (Figure 2B), CCoV-IIa ω = 0.22195 (Figure 2B), CCoV-IIb ω = 0.52488 (Figure 2B), and CCoV-IIv ω = 0.00148 (Figure 2B). The statistical *P*-value of all analyses was significant (*p* < 0.05). Two FCoV subtypes were subject to negative selection, but FCoV-II was subject to stronger constraints than FCoV-I. All subtypes of CCoV were constrained, and the order of the strength from strong to weak was CCoV-I, CCoV-IIv, CCoV-IIa, and CCoV-IIb. Interestingly, when analyzing CCoV-IIb from different hosts, we found that the canine-derived CCoV-IIb group was in negative selection (ω = 0.05935) (Figure 2C), while the human-derived CCoV-IIb group was in strong positive selection (ω = 20.89251) (Figure 2C). This result might be related to the sample size.

**FIGURE 3:**
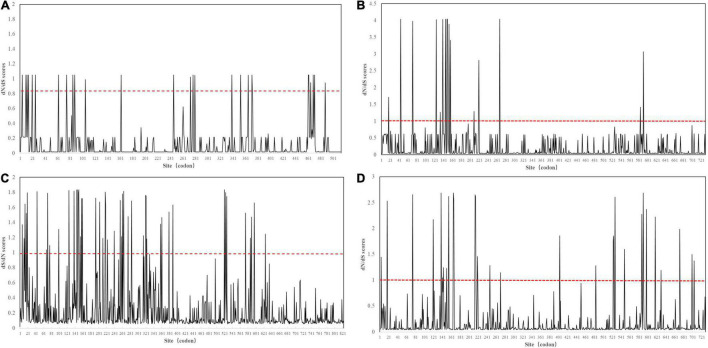
Sliding window analysis of S1 amino acid sites within each lineage. The red dotted line represents the critical value of dN/dS = 1. **(A)** FCoV-I. **(B)** FCoV-II. **(C)** CCoV-IIa. **(D)** CCoV-IIb.

Then, we applied the site model in EasyCodeML to analyze positive selection in each intra-lineage with a sufficient sample size. The *P*-value of all positive selection analyses was also significant (*p* < 0.05) (Supplementary Table 11). Nine positive sites were detected in FCoV-I subtype (reference: FCoV black, EU186072) (Table 1). Three positive sites were detected in FCoV-II subtype (reference: FCoV 79-1146, AY994055) (Table 1). We detected nine and ten positive sites in CCoV-IIa (reference: CCoV strain 171, KC175339) and CCoV-IIb (reference: CCoV UCD-1 AF116248), respectively (Table 1). The positive sites of FCoV-I and CCoV-IIb were more evenly distributed in the S1 gene, and those of FCoV-II and in CCoV-IIa group were mainly located in the N-terminal domain, the receptor-binding domain (Figure 3). The positive sites 786, 787, and 799 of FCoV-I were located on both sides of the S1/S2 recognition sequence “RRXRR,” which could be related to the cleavage of S protein during the cell invasion process of the virus. Each positive selection site had high variability and the properties of variable amino acid residues were different, which could help the virus to face different living environments (Table 1).

**TABLE 1:** Positive selection analysis of intra-lineage.

Strain	Positive Site	Positive Residue	P value	Variable Residue
FCoV-I	13	R	1.000**	G/H/K/L/M/N/R/S/T/
	25	Q	0.961*	H/K/P/Q/R/S/Y
	313	R	0.960*	K/N/R/S/T
	340	L	1.000**	A/D/H/I/K/L/P/S/V/Y
	472	Q	1.000**	E/K/N/Q/R/S
	560	K	0.991**	E/G/K/Q/R
	786	Q	1.000**	D/H/P/Q/R/S/T/W/Y/
	787	A	0.955*	A/H/L/P/S/T/V
	799	T	1.000**	H/K/N/Q/S/T
FCoV-II	148	Q	0.981*	D/K/Q/R/S/T
	152	N	0.990**	A/D/K/N/Q/S
	527	S	0.953*	I/M/S/V
CCoV-IIa	46	S	1.000**	H/Q/S/N
	73	R	0.982*	A/H/I/P/Q/R/T
	127	Q	0.987*	F/G/H/N/Q/R/Y
	142	R	0.997**	D/E/K/R/S/T
	148	Q	1.000**	A/L/M/Q/R/S/T
	151	S	1.000**	A/I/S/T/V
	152	N	1.000**	A/D/N/Q/R/S
	156	S	0.965*	A/D/H/N/S
	270	S	1.000**	A/E/P/S/V
CCoV-IIb	18	N	0.953*	D/E/N
	75	S	0.984*	D/N/R/S
	139	H	0.999**	H/K/T
	156	N	0.978*	N/P/Q
	166	S	0.999**	I/M/R/S/T
	167	Q	0.959*	A/D/E/G/Q/V
	215	L	0.983*	A/L/S
	216	Q	0.973*	A/E/Q/T
	529	T	0.970*	T/A/S
	592	Q	0.998**	Q/R/S

Statistically supported sites are marked with asterisks: *0.95 < P < 0.99; **P > 0.99.

### Phylogenetic Analysis and Evolutionary History of FCoV and CCoV

**FIGURE 4:**
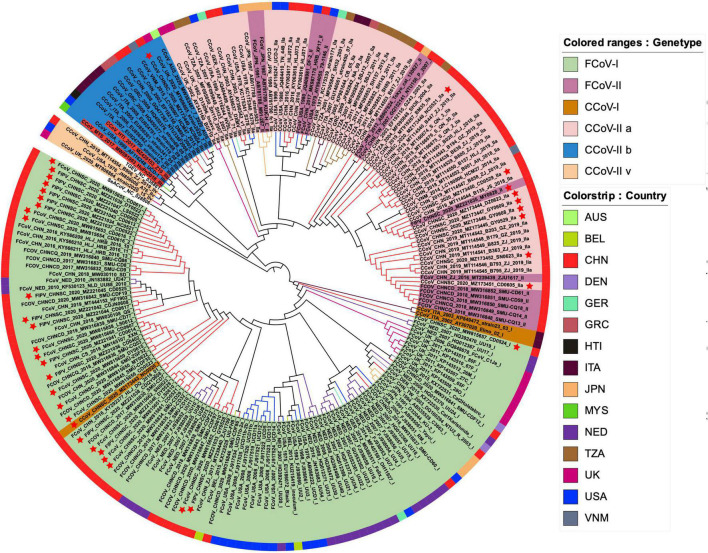
The maximum likelihood evolutionary tree of FCoV and CCoV S1 genes. The ML tree was reconstructed by IQTREE (version 2.1.3) in PhyloSuite (version 1.2.2) with GTR + F + R10 substitution model and 10,000 ultrafast bootstrap replicates. Genotypes are distinguished by color range. The color strip and clade color represent the geographic origin of the strain. The red star represents our sample. The name highlighted in red is human-derived CCoV. Each strain is named in the form of species–region–time–accession ID–strain–genotype.

We identified 37 S1 genes from our samples, of which 27 strains were clustered into FCoV-I subtype, eight strains were clustered into CCoV-IIa subtype, and only one FCoV-II strain and one CCoV-IIb strain were recognized (Figure 4). All FCoV-I strains and CCoV-I strains in our study were clustered in the same branch as the reference strains, such as UU strain from Netherlands and other strains from China (Figure 4). The FCoV-II and CCoV-IIa strains of our samples were clustered together with the Vietnamese strain CCoV/dog/HCM27/2014, the Tanzanian strain SH36_2004, and the Japanese strain Tokyo/cat/130627 (Figure 4). The only CCoV-IIb strain NC0604 was clustered together with the British CCoV strain 2020/7 and the Australian CCoV strain CCoV/7/2020/AUS, but it diverged from the human-derived CCoV (Figure 4). The FCoV strains from Taiwan, China were clustered together with the Japanese strains and showed certain genetic differences from the strains in the Chinese mainland (Figure 4).

According to the phylogeny results of N genes, most FCoV-I strains were clustered together with the Dutch strains, while most CCoV strains and Vietnamese strains were clustered together (Supplementary Figure 5). Their certain species characteristics could assist in species identification. Unlike the S1 genes and N genes, the M genes of FCoV and CCoV have an obvious connection (Supplementary Figure 6). In general, part of the M genes identified from our FCoV-positive samples was clustered with strains from the United States and Brazil. The M genes of seven strains were still in the same branch as the Dutch strains. Interestingly, there were five samples in a separate clade clustered with the Japanese strain FCoV Tokyo/cat/130627. Most of the CCoV M genes were clustered with the Chinese strains.

**FIGURE 5:**
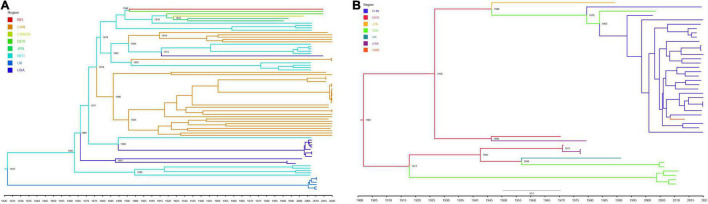
Maximum clade credibility tree of FCoV-I **(A)** and CCoV-IIa **(B)** S1 gene. Viruses from different regions are denoted by different colors. Some important dates are marked on the branch nodes. **(A)** MCC tree of FCoV-I S1 gene was reconstructed by the strict clock model with the Bayesian Skygrid coalescent model and four independent chains with a chain length of 1 × 10^8^ using BEAST (version 1.10.4). **(B)** MCC tree of CCoV-IIa S1 gene was reconstructed by one chain with a chain length of 5 × 10^7^ for CCoV-IIa under the uncorrelated lognormal relaxed clock model with the Bayesian skyline coalescent model using BEAST (version 1.10.4).

Due to the limitations of the number of sequences, we only performed phylodynamic analysis on the S1 gene of the classical strains from FCoV-I and CCoV-IIa subtypes. The dataset had an optimal temporal signal (FCoV R^2^ = 0.5816, CCoV R^2^ = 0.706) using TempEst (Supplementary Figure 7). Further BETS analysis results showed that log BF(FCoV) = 2400.29 > 5 and log BF(CCoV) = 19.439 > 5, so Mhet was better than Miso and the dataset had an excellent temporal signal (Supplementary Table 12). The MCC trees showed the temporal historical origin of FCoV and CCoV based on the S1 gene. The mean evolutionary rate of FCoV-I S1 genes was estimated at 1.244 × 10^–3^ (95% HPD: 1.13 × 10^–3^−1.34 × 10^–3^) subs/site/year. The time of the most recent common ancestor (tMRCA) of FCoV-I was estimated at 1822 (95% HPD: 1801–1841), which was probably the origin time of the British FCoV population and time of differentiation with the Netherlandish population (Figure 5A). The estimated tMRCA of the Netherlandish FCoV-I groups was 1862 (95% HPD: 1855–1876), and that of the United States population was 1887 (95% HPD: 1876–1899) (Figure 5A). We also estimated that the earliest origin of the Chinese FCoV-I population was 1876 (95% HPD: 1866–1884) and the tMRCA of the Chinese population was 1886 (95% HPD: 1876–1892) (Figure 5A). Then, the mean evolutionary rate of CCoV-IIa S1 gene was estimated at 1.281 × 10^–3^ (95% HPD: 9.40 × 10^–4^−1.63 × 10^–3^) subs/site/year. The tMRCA of CCoV-IIa was 1901 (95% HPD: 1870–1929) derived from the German CCoV population (Figure 5B). The origin time of CCoV-IIa of Tanzania was 1917 (95% HPD: 1886–1946) (Figure 5B). The CCoV-IIa of a few Chinese strains was originated in 1979 (95% HPD: 1968–1991), but we could clearly find that the origin time of most Chinese strains in the dataset was 1983 (95% HPD: 1975–1993) (Figure 5B). By combining the estimation of the S1 genes of FCoV-I and CCoV-II, we concluded that the tMRCA of FCoV and CCoV was 4,598 years ago, which is 2578 BC (95% HPD: 4808 BC–500 BC) (Supplementary Figure 8).

**FIGURE 6:**
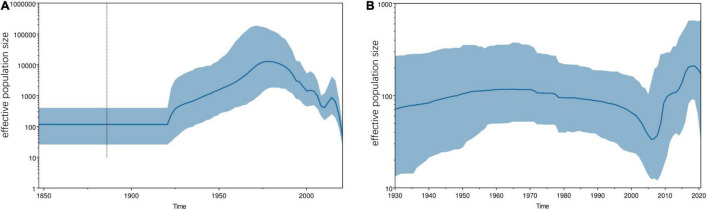
Demographic history of FCoV-I **(A)** and CCoV-IIa **(B)**. Plots show the effective population size (y-axis) through time (x-axis). The blue lines indicate the median estimates of effective population size, and the shaded regions indicate the corresponding 95% confidence interval. **(A)** FCoV-I demographic history was reconstructed by the Bayesian Skygrid coalescent model. **(B)** CCoV-IIa demographic history was reconstructed by the Bayesian skyline coalescent model.

The analysis of population historical dynamics shows that the FCoV population was in a stable state before 1920 (Figure 6A). After that, the population size began to expand rapidly. However, it began to shrink around 1975 (Figure 6). Although the population size fluctuated slightly after 2000, the overall trend was shrinking at a historically low level (Figure 6A). The CCoV population size had grown slowly before 1970. After that, it has begun to shrink slowly (Figure 6B). However, it began to shrink rapidly in 2000 and expand sharply in 2005 (Figure 6B). Although there was a slight decline thereafter, the overall population size was at a historically high level.

## Discussion

Canine coronavirus and FCoV, as coronaviruses carried by companion animals, were threats not only to dogs and cats but also to public health. Although the recombination and genetic characteristics of CCoV and FCoV had been reported in many studies in other regions, they are mostly independent analyses (Terada et al., 2014). Such studies were still lacking in China, especially in terms of phylodynamics. Our research showed that FCoV-I strains and CCoV-IIa strains were mainly prevalent in Sichuan, while FCoV-II and CCoV-IIb recombinant strains also existed in this area (Figure 4). Sequence alignment showed that the S1 gene of the recombinant strain was different from the classic strain, which increased the genetic diversity to a certain extent. Population genetic analysis showed that the FCoV population in Sichuan was seriously differentiated from the American and British populations but was moderately differentiated from the Dutch population. The analysis results indicated that the Sichuan FCoV population might be in an independent direction of differentiation (Supplementary Table 9). The population structure could show the distribution of the same genetic characteristics among different geographic groups. DAPC analysis showed that the S1, N, and M genes of Sichuan FCoV contained the same genetic components as most Dutch FCoV groups and that as a small number of American and Japanese FCoV populations (Supplementary Figures 1–3). Secondly, the scatter plot also showed that the genetic components of Sichuan FCoV groups had significant crossovers with Netherlandish, American, and Japanese FCoV groups, having a genetic link (Supplementary Figure 4). In addition, the phylogeny of the S1 genes showed that many S1 genes identified from our FCoV-positive samples were clustered in one clade with the Dutch UU strain series (Figure 4). The results above indicated that the FCoV groups of Sichuan and Netherlands might have a common ancestor. However, due to the sample size of CCoV, population genetic analysis for CCoV was not performed, but the phylogeny showed that Sichuan CCoV had a homologous relationship with strains from Vietnam, Tanzania, and Japan (Figure 4). Although we tried to reveal the relationship between FIPV, FECV, and CCoV through the phylogeny of N and M genes, it was obvious that these two genes cannot be used as molecular markers to distinguish them. On the contrary, the N gene can distinguish FCoV and CCoV more clearly than the M gene, which could assist the S gene in the rapid identification of virus species.

As we know, genetic recombination could promote the generation of the new lineage, adapt to the host, and expand the host range (Herrewegh et al., 1998). Coronavirus had a low-fidelity polymerase and a unique replication-selection mechanism, henceforth having a high recombination frequency (Smith and Denison, 2012). A previous study had indicated that CCoV and FCoV had high intra-lineage and inter-lineage recombination, which was also the reason for the emergence of FCoV-II and CCoV-I (Le Poder, 2011). Interestingly, CCoV-IIb was derived from the S1 gene recombination of CCoV-IIa and TGEV (Regan et al., 2012). Our research found that the S1 gene had the most frequent recombination, followed by the M gene. The recombination frequency of CCoV was higher than that of FCoV, while there was no significant difference within the inter-lineage. We had also detected CCoV-IIb produced by interspecific recombination in our samples using the same recombination method. The assumed breakpoint was the same as in previous studies. Then, the previous study claimed that a few CCoV-IIb strains were isolated from humans and we indicated that human-derived CCoV-IIb was in strong positive selection, so this should arouse our attention to the emergence of recombinant strains to monitor its adaptation to the human host (Lednicky et al., 2021; Vlasova et al., 2021). Most of the recombination events we found occurred in the N-terminal domain of the S1 gene. Coincidentally, most of the sites with ω > 1 and more non-synonymous amino acid mutations were also located in this region. Although this region was not the protein receptor-binding domain of CCoV and FCoV, the positive selection site located in this region may be a key position for regulating virus entry into cells and immune evasion.

The average evolution rates of FCoV S1 and CCoV S1 genes were 1.244 × 10^–3^ subs/site/year (95% HPD: 1.13 × 10^–3^−1.34 × 10^–3^) and 1.281 × 10^–3^ subs/site/year (95% HPD: 9.40 × 10^–3^–1.63 × 10^–3^), respectively, which were far lower than the evolution rate of PEDV reported in previous studies of 2.22 × 10^–2^ subs/site/year (Sung et al., 2015) and the evolution rate of PDCoV of 1.67 × 10^–3^ subs/site/year (He et al., 2020). This situation was somewhat similar to the previous description of the lack of FCoV and CCoV vaccines on a large scale and most animals carrying the virus were healthy, so both the pressure from vaccination and natural infection were small (He et al., 2020). However, PEDV vaccines were widely used in farms and the long-term pressure on the immune system has forced the evolution of PEDV to accelerate. The phenomenon that the evolution rate of PDCoV was higher than that of FCoV and CCoV could have resulted in the huge number of pig breeding and the existence of multiple breeding modes and trading chains, which provide more opportunities for the virus to spread and evolve. The evolution rate of CCoV was higher than that of FCoV. The population history showed that the population size of CCoV was at a high level, while that of FCoV was at a low level. Combined with selection pressure, the evolutionary potential of CCoV was even greater, especially CCoV-IIb.

In summary, our research provided a comprehensive understanding of CCoV and FCoV evolution and a molecular epidemiological assessment of their temporal origins. We found that CCoV-IIb strains were in positive selection, which had an important vigilant concern for public health. Recurrent epidemiological surveillance for coronavirus infections among cats and dogs is needed to get a better insight into a detailed evolutionary trend of pathogens and transmission dynamics, which could also serve as an early cautioning system for human and animal threats.

## Data Availability Statement

The original contributions presented in the study are included in the article/Supplementary Material, further inquiries can be directed to the corresponding author/s.

## Author Contributions

HY and QP carried out the experiments. SZ and QY supervised the study. YL and SZ contributed to the conception of this article. HY, QP, YL, SD, SC, RW, QZ, XH, YW, JL, SZ, and QY analyzed the data. All authors reviewed the manuscript.

## Conflict of Interest

The authors declare that the research was conducted in the absence of any commercial or financial relationships that could be construed as a potential conflict of interest.

## Publisher’s Note

All claims expressed in this article are solely those of the authors and do not necessarily represent those of their affiliated organizations, or those of the publisher, the editors and the reviewers. Any product that may be evaluated in this article, or claim that may be made by its manufacturer, is not guaranteed or endorsed by the publisher.

## Footnotes

1
https://itol.embl.de/
