# Supplementary material for: Phylogeny, Evolution, and Transmission Dynamics of Canine and Feline Coronaviruses: A Retro-Prospective Study
Source: Front Microbiol. 2022 Apr 26;13:850516. doi: 10.3389/fmicb.2022.850516 (PMC9087556; doi:10.3389/fmicb.2022.850516)
Supplement: Supplementary file 1 [file Data_Sheet_1.docx]

Supplementary Material

# Supplementary Figures and Tables

Table S1. All S1 gene and partial M and N gene sequence information used in this study.

| Strain | Location | Code Letter | Date | Host | Gene Accession Number | | | Source |
| --- | --- | --- | --- | --- | --- | --- | --- | --- |
|  |  |  |  |  | S | M | N |  |
| FECV MY0622 | China Sichuan | CHN | 20200620 | Feline | MW815650 | MW722859 | MW722825 | This study |
| FECV LS0610 | China Sichuan | CHN | 20200610 | Feline | MW815651 | MW722860 | MW722826 | This study |
| FECV CD0610 | China Sichuan | CHN | 20200610 | Feline | MW815652 | MW722861 | MW722827 | This study |
| FECV CD0616-1 | China Sichuan | CHN | 20200616 | Feline | MW815653 | MW722862 | MW722828 | This study |
| FECV CD0616-2 | China Sichuan | CHN | 20200616 | Feline | MW815654 | MW722863 | MW722829 | This study |
| FECV CD0617 | China Sichuan | CHN | 20200617 | Feline | MW815655 | MW722864 | MW722830 | This study |
| FECV CD0523 | China Sichuan | CHN | 20200523 | Feline | MW815656 | MW722865 | MW722831 | This study |
| FECV CD0524 | China Sichuan | CHN | 20200524 | Feline | MW815657 | MW722866 | MW722832 | This study |
| FECV LS0612 | China Sichuan | CHN | 20200612 | Feline | MW815658 | MW722867 | MW722833 | This study |
| FECV DY0615 | China Sichuan | CHN | 20200615 | Feline | MW815659 | MW722868 | MW722834 | This study |
| FECV CD0607 | China Sichuan | CHN | 20200607 | Feline | MW815660 | MW722869 | MW722835 | This study |
| FECV CD0521 | China Sichuan | CHN | 20200521 | Feline | MW815661 | MW722870 | MW722836 | This study |
| FECV SN0623 | China Sichuan | CHN | 20200623 | Feline | MW815662 | MW722871 | MW722837 | This study |
| FIPV GY0528 | China Sichuan | CHN | 20200528 | Feline | MZ221033 | MW722872 | MW722838 | This study |
| FIPV NC0521 | China Sichuan | CHN | 20200521 | Feline | MZ221034 | MW722873 | MW722839 | This study |
| FIPV MY0628 | China Sichuan | CHN | 20200628 | Feline | MZ221035 | MW722874 | MW722840 | This study |
| FIPV CD0402 | China Sichuan | CHN | 20200402 | Feline | MZ221036 | MW722875 | MW722841 | This study |
| FIPV CD0522 | China Sichuan | CHN | 20200522 | Feline | MZ221037 | MW722876 | MW722842 | This study |
| FIPV CDQY0608 | China Sichuan | CHN | 20200608 | Feline | MZ221038 | MW722877 | MW722843 | This study |
| FIPV LS0526 | China Sichuan | CHN | 20200526 | Feline | NA | MW722878 | MW722844 | This study |
| FIPV LS0530 | China Sichuan | CHN | 20200530 | Feline | NA | MW722879 | MW722845 | This study |
| FIPV LS0606 | China Sichuan | CHN | 20200606 | Feline | NA | MW722880 | MW722846 | This study |
| FIPV DY0522 | China Sichuan | CHN | 20200522 | Feline | MZ221039 | MW722881 | MW722847 | This study |
| FIPV DY0523 | China Sichuan | CHN | 20200523 | Feline | NA | MW722882 | MW722848 | This study |
| FIPV CD0609 | China Sichuan | CHN | 20200609 | Feline | MZ221040 | MW722883 | MW722849 | This study |
| FIPV CD0614 | China Sichuan | CHN | 20200614 | Feline | MZ221041 | MW722884 | MW722850 | This study |
| FIPV CDJN0608 | China Sichuan | CHN | 20200608 | Feline | MZ221042 | MW722885 | MW722851 | This study |
| FIPV CD0613 | China Sichuan | CHN | 20200613 | Feline | MZ221043 | MW722886 | MW722852 | This study |
| FIPV CD0615 | China Sichuan | CHN | 20200615 | Feline | NA | MW722887 | MW722853 | This study |
| FIPV CD0617 | China Sichuan | CHN | 20200617 | Feline | MZ221044 | MW722888 | MW722854 | This study |
| FIPV CD0525 | China Sichuan | CHN | 20200525 | Feline | MZ221045 | MW722889 | MW722855 | This study |
| FIPV DY0528 | China Sichuan | CHN | 20200528 | Feline | MZ221046 | MW722890 | MW722856 | This study |
| FIPV DY0612 | China Sichuan | CHN | 20200612 | Feline | NA | MW722891 | MW722857 | This study |
| FIPV LS0522 | China Sichuan | CHN | 20200522 | Feline | MZ221047 | MW722892 | MW722858 | This study |
| CCoV NC0520 | China Sichuan | CHN | 20200520 | Canine | MZ173443 | MW718807 | MW718795 | This study |
| CCoV LS0611 | China Sichuan | CHN | 20200520 | Canine | NA | MW718808 | MW718796 | This study |
| CCoV DZ0623 | China Sichuan | CHN | 20200623 | Canine | MZ173444 | MW718809 | MW718797 | This study |
| CCoV GY0529 | China Sichuan | CHN | 20200529 | Canine | MZ173445 | MW718810 | MW718798 | This study |
| CCoV GY0608 | China Sichuan | CHN | 20200608 | Canine | MZ173446 | MW718811 | MW718799 | This study |
| CCoV GY0609 | China Sichuan | CHN | 20200609 | Canine | MZ173447 | MW718812 | MW718800 | This study |
| CCoV NC0521 | China Sichuan | CHN | 20200521 | Canine | MZ173448 | MW718813 | MW718801 | This study |
| CCoV NC0604 | China Sichuan | CHN | 20200604 | Canine | MZ173449 | MW718814 | MW718802 | This study |
| CCoV CD0529 | China Sichuan | CHN | 20200529 | Canine | MZ173450 | MW718815 | MW718803 | This study |
| CCoV CD0605 | China Sichuan | CHN | 20200605 | Canine | MZ173451 | MW718816 | MW718804 | This study |
| CCoV SN0628 | China Sichuan | CHN | 20200628 | Canine | NA | MW718817 | MW718805 | This study |
| CCoV SN0623 | China Sichuan | CHN | 20200623 | Canine | MZ173452 | MW718818 | MW718806 | This study |
| FIPV 79-1146 | United States | USA | 1979 | Feline | AY994055，complete genome | | | GenBank |
| FIPV C1Je | United Kingdom | UK | 2006 | Feline | DQ848678，complete genome | | | GenBank |
| FIPV Black | United States | USA | 1975 | Feline | EU186072，complete genome | | | GenBank |
| FCoV RM | USA: California | USA | 20020128 | Feline | FJ938051，complete genome | | | GenBank |
| FCoV UU11 | Netherlands | NED | 20070605 | Feline | FJ938052，complete genome | | | GenBank |
| FCoV UU7 | Netherlands | NED | 20070425 | Feline | FJ938053，complete genome | | | GenBank |
| FIPV UU4 | Netherlands | NED | 20070202 | Feline | FJ938054，complete genome | | | GenBank |
| FIPV UU8 | Netherlands | NED | 20070524 | Feline | FJ938055，complete genome | | | GenBank |
| FIPV UU5 | Netherlands | NED | 20070307 | Feline | FJ938056，complete genome | | | GenBank |
| FIPV UU15 | Netherlands | NED | 20070713 | Feline | FJ938057，complete genome | | | GenBank |
| FIPV UU16 | Netherlands | NED | 20070607 | Feline | FJ938058，complete genome | | | GenBank |
| FCoV UU10 | Netherlands | NED | 20070605 | Feline | FJ938059，complete genome | | | GenBank |
| FCoV UU2 | USA: California | USA | 19930330 | Feline | FJ938060，complete genome | | | GenBank |
| FIPV UU3 | USA: California | USA | 19980310 | Feline | FJ938061，complete genome | | | GenBank |
| FIPV UU9 | Netherlands | NED | 20070524 | Feline | FJ938062，complete genome | | | GenBank |
| FCoV/NTU156/P | China Taiwan | CHN_TW | 200709 | Feline | GQ152141，complete genome | | | GenBank |
| FCoV UU22 | Netherlands | NED | 20070913 | Feline | GU553361，complete genome | | | GenBank |
| FCoV UU23 | Netherlands | NED | 20070913 | Feline | GU553362，complete genome | | | GenBank |
| FIPV UU17 | Netherlands | NED | 20070717 | Feline | HQ012367，complete genome | | | GenBank |
| FCoV UU18 | Netherlands | NED | 20070724 | Feline | HQ012368，complete genome | | | GenBank |
| FIPV UU21 | Netherlands | NED | 20070911 | Feline | HQ012369，complete genome | | | GenBank |
| FIPV UU24 | Netherlands | NED | 20080103 | Feline | HQ012370，complete genome | | | GenBank |
| FCoV UU31 | Netherlands | NED | 20080125 | Feline | HQ012371，complete genome | | | GenBank |
| FCoV UU34 | Netherlands | NED | 20070911 | Feline | HQ012372，complete genome | | | GenBank |
| FCoV UU40 | Netherlands | NED | 20080122 | Feline | HQ392469，complete genome | | | GenBank |
| FCoV UU19 | Netherlands | NED | 20070823 | Feline | HQ392470，complete genome | | | GenBank |
| FCoV UU20 | Netherlands | NED | 20070823 | Feline | HQ392471，complete genome | | | GenBank |
| FIPV UU30 | Netherlands | NED | 20080122 | Feline | HQ392472，complete genome | | | GenBank |
| FCoV UU47 | Netherlands | NED | 20100714 | Feline | JN183882，complete genome | | | GenBank |
| FCoV UU54 | Netherlands | NED | 20100312 | Feline | JN183883，complete genome | | | GenBank |
| FIPV DF-2 | United States | USA | 1980 | Feline | JQ408981，complete genome | | | GenBank |
| FCoV UU88 | Netherlands | NED | 20100817 | Feline | KF530123，complete genome | | | GenBank |
| FIPV isolate 27C | United Kingdom | UK | 20110119 | Feline | KP143507，complete genome | | | GenBank |
| FIPV isolate 28O | United Kingdom | UK | 20110204 | Feline | KP143508，complete genome | | | GenBank |
| FCoV isolate 65F | United Kingdom | UK | 201101 | Feline | KP143509，complete genome | | | GenBank |
| FCoV isolate 67F | United Kingdom | UK | 201101 | Feline | KP143510，complete genome | | | GenBank |
| FCoV isolate 80F | United Kingdom | UK | 201101 | Feline | KP143511，complete genome | | | GenBank |
| FIPV isolate 26M | United Kingdom | UK | 20110129 | Feline | KP143512，complete genome | | | GenBank |
| FCoV inoculum | Belgium | BEL | 2013 | Feline | KU215419，complete genome | | | GenBank |
| FCoV Cat1_day7 | Belgium | BEL | 2013 | Feline | KU215420，complete genome | | | GenBank |
| Cat2_day21_deletion | Belgium | BEL | 2013 | Feline | KU215421，complete genome | | | GenBank |
| FCoV Cat2_day21_w_d | Belgium | BEL | 2013 | Feline | KU215422，complete genome | | | GenBank |
| FCoV Cat3_day9 | Belgium | BEL | 2013 | Feline | KU215423，complete genome | | | GenBank |
| FCoV Cat1_day28_d | Belgium | BEL | 2013 | Feline | KU215424，complete genome | | | GenBank |
| FCoV Cat1day28_ w_d | Belgium | BEL | 2013 | Feline | KU215425，complete genome | | | GenBank |
| FCoV Cat2_day84 | Belgium | BEL | 2013 | Feline | KU215426，complete genome | | | GenBank |
| FCoV Cat3day28_d | Belgium | BEL | 2013 | Feline | KU215427，complete genome | | | GenBank |
| FCoV Cat3day28_ w_d | Belgium | BEL | 2013 | Feline | KU215428，complete genome | | | GenBank |
| FCoV UG-FH8 | Belgium | BEL | 20150101 | Feline | KX722529，complete genome | | | GenBank |
| FIPV Cat 1 Karlslunde | Denmark | DEN | 20150201 | Feline | KX722530，complete genome | | | GenBank |
| FIPV HLJ/DQ/2016/01 | China | CHN | 201610 | Feline | KY292377，complete genome | | | GenBank |
| FIPV HLJ/HRB/2016/10 | China | CHN | 201611 | Feline | KY566209，complete genome | | | GenBank |
| FIPV HLJ/HRB/2016/11 | China | CHN | 201611 | Feline | KY566210，complete genome | | | GenBank |
| FIPV HLJ/HRB/2016/13 | China | CHN | 201611 | Feline | KY566211，complete genome | | | GenBank |
| FCoV Felix | Germany | GER | 201207 | Feline | MG893511，complete genome | | | GenBank |
| FCoV isolate XXN | China | CHN | 201804 | Feline | MN165107，complete genome | | | GenBank |
| FIPV ZJU1617 | China Zhejiang | CHN | 2016 | Feline | MT239439，complete genome | | | GenBank |
| FIPV ZJU1709 | China Zhejiang | CHN | 201709 | Feline | MT239440，complete genome | | | GenBank |
| FIPV HF1902 | China | CHN | 20190525 | Feline | MT444152，complete genome | | | GenBank |
| FCoV isolate QS | China | CHN | 201805 | Feline | MW030108，complete genome | | | GenBank |
| FCoV isolate SD | China | CHN | 201805 | Feline | MW030110，complete genome | | | GenBank |
| CCoV strain 23/03 | Italy | ITA | 2003 | Canine | KP849472，complete genome | | | GenBank |
| CCoV/NTU336/F/2008 | China Taiwan | CHN_TW | 200811 | Canine | GQ477367，complete genome | | | GenBank |
| CCoV strain A76 | United States | USA | 1976 | Canine | JN856008，complete genome | | | GenBank |
| CCoV strain 1-71 | Germany | GER | 1972 | Canine | JQ404409，complete genome | | | GenBank |
| CCoV strain TN-449 | United States | USA | 1980 | Canine | JQ404410，complete genome | | | GenBank |
| CCoV strain 171 | Germany | GER | 1971 | Canine | KC175339，complete genome | | | GenBank |
| CCoV strain K378 | United States | USA | 1978 | Canine | KC175340，complete genome | | | GenBank |
| CCoV strain S378 | United States | USA | 1978 | Canine | KC175341，complete genome | | | GenBank |
| CCoV strain CB/05 | Italy | ITA | 2005 | Canine | KP981644，complete genome | | | GenBank |
| CCoV HLJ-071 | China | CHN | 20160701 | Canine | KY063616，complete genome | | | GenBank |
| CCoV HLJ-072 | China | CHN | 20160701 | Canine | KY063617，complete genome | | | GenBank |
| CCoV HLJ-073 | China | CHN | 20160701 | Canine | KY063618，complete genome | | | GenBank |
| CCoV strain 2020/15 | United Kingdom | UK | 2020 | Canine | MT906864，complete genome | | | GenBank |
| CCoV strain 2020/7 | United Kingdom | UK | 2020 | Canine | MT906865，complete genome | | | GenBank |
| CCoV-HuPn-2018 | Malaysia | MYS | 2017 | Homo sapiens | MW591993，complete genome | | | GenBank |
| CCoV strain Z19 | Haiti | HTI | 2017 | Homo sapiens | MZ420153，complete genome | | | GenBank |
| FIPV UCD1 | United States | USA | 1976 | Feline | AB088222，S gene CDS | | | GenBank |
| FCoV C3663 | Japan | JPN | 1994 | Feline | AB535528，S gene CDS | | | GenBank |
| FCoV Yayoi | Japan: Tokyo | JPN | 1991 | Feline | AB695067，S gene CDS | | | GenBank |
| FCoV M91-267 | Japan | JPN | 1991 | Feline | AB781788，S gene CDS | | | GenBank |
| FCoV KUK-H/L | Japan | JPN | 1987 | Feline | AB781789，S gene CDS | | | GenBank |
| FCoV Tokyo/cat/130627 | Japan:Tokyo | JPN | 2013 | Feline | AB907624，S gene CDS | | | GenBank |
| FCoV KU-2 | Japan | JPN | 1991 | Feline | D32044，S gene CDS | | | GenBank |
| FCoV/NTU2/R/2003 | China Taiwan | CHN_TW | 2003 | Feline | DQ160294，S gene CDS | | | GenBank |
| FIPV-UCD11a | United States | USA | 20080131 | Feline | FJ917519，S gene CDS | | | GenBank |
| FIPV-UCD11b | United States | USA | 20080131 | Feline | FJ917520，S gene CDS | | | GenBank |
| FIPV-UCD12 | United States | USA | 20080212 | Feline | FJ917521，S gene CDS | | | GenBank |
| FECV-UCD5 | United States | USA | 20080212 | Feline | FJ917522，S gene CDS | | | GenBank |
| FIPV-UCD13 | United States | USA | 20080117 | Feline | FJ917523，S gene CDS | | | GenBank |
| FIPV-UCD14 | United States | USA | 20070511 | Feline | FJ917524，S gene CDS | | | GenBank |
| FIPV-UCD11b-2a | United States | USA | 20080404 | Feline | FJ917534，S gene CDS | | | GenBank |
| FIPV-UCD11b-2b | United States | USA | 20080404 | Feline | FJ917535，S gene CDS | | | GenBank |
| FCoV Cat 2 Holstebro | Denmark | DEN | 20150201 | Feline | KX722531，S gene CDS | | | GenBank |
| FCoV OH11927 | USA: Ohio | USA | 20141126 | Feline | MF457591，S gene CDS | | | GenBank |
| FCoV HRB/XF17 | China | CHN | 20170716 | Feline | MK987175，S gene CDS | | | GenBank |
| CCoV strain5821 | Japan | JPN | 1997 | Canine | AB017789，S gene CDS | | | GenBank |
| CCoV fc1 | Japan | JPN | 1990 | Canine | AB781790，S gene CDS | | | GenBank |
| CCoV Elmo_02 | Italy | ITA | 2002 | Canine | AY307020，S gene CDS | | | GenBank |
| CCoV BGF10 | United Kingdom | UK | 2002 | Canine | AY342160，S gene CDS | | | GenBank |
| CCoV v1 | China | CHN |  | Canine | AY390342，S gene CDS | | | GenBank |
| CCoV DXMV | China | CHN | 2003 | Giant panda | AY436637，S gene CDS | | | GenBank |
| CCoV Insavc_1 | United Kingdom | UK |  | Canine | D13096，S gene CDS | | | GenBank |
| CCoV GZ43/2003 | China | CHN | 2003 | Raccoon dog | EF192155，S gene CDS | | | GenBank |
| CCoV DM95/2003 | China | CHN | 2003 | Ferret badger | EF192156，S gene CDS | | | GenBank |
| CCoV strain341/05 | Italy | ITA | 200512 | Canine | EU856361，S gene CDS | | | GenBank |
| CCoV strain174/06 | Italy | ITA | 200603 | Canine | EU856362，S gene CDS | | | GenBank |
| CCoV strain430/07 | Italy | ITA | 200710 | Canine | EU924790，S gene CDS | | | GenBank |
| CCoV strain119/08 | Italy | ITA | 200803 | Canine | EU924791，S gene CDS | | | GenBank |
| CCoV strain450/07 | Italy | ITA | 200710 | Canine | GU146061，S gene CDS | | | GenBank |
| CCoV strain66/09 | Greece | GRC | 2009 | Canine | HQ450376，S gene CDS | | | GenBank |
| CCoV strain68/09 | Greece | GRC | 2009 | Canine | HQ450377，S gene CDS | | | GenBank |
| CCoV NA/09 | Greece | GRC | 2009 | Canine | JF682842，S gene CDS | | | GenBank |
| CCoV HCM27/2014 | Viet Nam | VNM | 201401 | Canine | LC190906，S gene CDS | | | GenBank |
| CCoV HCM47/2015 | Viet Nam | VNM | 201506 | Canine | LC190907，S gene CDS | | | GenBank |
| CCoV SH32/2001 | Tanzania | TZA | 2001 | Crocuta crocuta | MF095847，S gene CDS | | | GenBank |
| CCoV SH36/2004 | Tanzania | TZA | 2004 | Crocuta crocuta | MF095848，S gene CDS | | | GenBank |
| CCoV SH33/2007 | Tanzania | TZA | 2007 | Crocuta crocuta | MF095849，S gene CDS | | | GenBank |
| CCoV SH110/2007 | Tanzania | TZA | 2007 | Crocuta crocuta | MF095850，S gene CDS | | | GenBank |
| CCoV SH89/2011 | Tanzania | TZA | 2011 | Crocuta crocuta | MF095851，S gene CDS | | | GenBank |
| CCoV SH143/2011 | Tanzania | TZA | 2011 | Crocuta crocuta | MF095852，S gene CDS | | | GenBank |
| CCoV SH157/2012 | Tanzania | TZA | 2012 | Crocuta crocuta | MF095853，S gene CDS | | | GenBank |
| CCoV SBJ12/2007 | Tanzania | TZA | 2007 | Canine | MF095854，S gene CDS | | | GenBank |
| CCoV SBJ3/2011 | Tanzania | TZA | 2011 | Canine | MF095855，S gene CDS | | | GenBank |
| CCoV B639/ZJ/2019 | China | CHN | 20190919 | Canine | MT114538，S gene CDS | | | GenBank |
| CCoV B600/ZJ/2019 | China | CHN | 20190915 | Canine | MT114539，S gene CDS | | | GenBank |
| CCoV B447/ZJ/2019 | China | CHN | 20190726 | Canine | MT114540，S gene CDS | | | GenBank |
| CCoV B363/ZJ/2019 | China | CHN | 20190709 | Canine | MT114541，S gene CDS | | | GenBank |
| CCoV B203/GZ/2019 | China | CHN | 20190102 | Canine | MT114542，S gene CDS | | | GenBank |
| CCoV B194/GZ/2019 | China | CHN | 20190127 | Canine | MT114543，S gene CDS | | | GenBank |
| CCoV B135/JS/2018 | China | CHN | 20191210 | Canine | MT114544，S gene CDS | | | GenBank |
| CCoV B795/ZJ/2019 | China | CHN | 20191105 | Canine | MT114545，S gene CDS | | | GenBank |
| CCoV B793/ZJ/2019 | China | CHN | 20191102 | Canine | MT114546，S gene CDS | | | GenBank |
| CCoV B858/ZJ/2019 | China | CHN | 20191107 | Canine | MT114547，S gene CDS | | | GenBank |
| CCoV B179/GZ/2019 | China | CHN | 20190127 | Canine | MT114548，S gene CDS | | | GenBank |
| CCoV B825/ZJ/2019 | China | CHN | 20190924 | Canine | MT114549，S gene CDS | | | GenBank |
| CCoV B157/HLJ/2019 | China | CHN | 20190111 | Canine | MT114550，S gene CDS | | | GenBank |
| CCoV B020/HLJ/2018 | China | CHN | 20180918 | Canine | MT114551，S gene CDS | | | GenBank |
| CCoV B001/AH/2018 | China | CHN | 20181107 | Canine | MT114552，S gene CDS | | | GenBank |
| CCoV B617/ZJ/2019 | China | CHN | 20190915 | Canine | MT114553，S gene CDS | | | GenBank |
| CCoV B906/ZJ/2019 | China | CHN | 20191210 | Canine | MT114554，S gene CDS | | | GenBank |
| CCoV S-QN-3 | China | CHN | 20180309 | Canine | MT166674，S gene CDS | | | GenBank |
| CCoV S-QN-2 | China | CHN | 20180309 | Canine | MT166675，S gene CDS | | | GenBank |
| CCoV S-QN-30 | China | CHN | 20180309 | Canine | MT166676，S gene CDS | | | GenBank |
| CCoV BM35 | China | CHN | 20191120 | Canine | MT919267，S gene CDS | | | GenBank |
| CCoV 7_2020_AUS | Australia | AUS | 20200312 | Canine | MW383487，S gene CDS | | | GenBank |
| CCoV strain NVSL | United States | USA | 19990813 | Canine | AF116244，S gene CDS | | | GenBank |
| CCoV strain UCD-2 | United States | USA | 19990813 | Canine | AF116247，S gene CDS | | | GenBank |
| CCoV strain UCD-1 | United States | USA | 19990813 | Canine | AF116248，S gene CDS | | | GenBank |

*NA，Not Available

Table S2. FCoV S1 Gene Sequence Identity Matrix

|  | MY0622 | LS0610 | CD0610 | **CD06161** | CD06162 | **CD0617** | **CD0523** | **CD0524** | LS0612 | DY0615 | CD0607 | CD0521 | SN0623 | GY0528 | MY0628 | CD0402 | CD0522 | CDQY68 | CD0614 | JN0608 | CD0525 | DY0528 |
| --- | --- | --- | --- | --- | --- | --- | --- | --- | --- | --- | --- | --- | --- | --- | --- | --- | --- | --- | --- | --- | --- | --- |
| MY0622 | - | 84.69  88.81 | 83.57  87.30 | 83.31  87.51 | 83.35  87.51 | 83.24  87.16 | 86.81  89.73 | 73.90  76.80 | 85.72  89.64 | 87.36  91.48 | 85.57  90.55 | 84.34  87.88 | 84.58  89.86 | 83.68  87.41 | 42.18  31.77 | 86.43  90.43 | 83.24  87.16 | 84.38  87.53 | 85.38  89.85 | 82.15  87.66 | 83.35  86.38 | 83.37  87.18 |
| LS0610 | 84.69  88.81 | - | 83.84  86.83 | 83.29  86.13 | 83.33  86.13 | 83.22  85.78 | 85.04  88.11 | 73.54  75.55 | 83.72  87.44 | 85.66  88.46 | 85.20  89.16 | 88.54  91.84 | 89.20  92.77 | 83.95  86.95 | 42.58  31.66 | 84.81  88.00 | 83.22  85.78 | 84.75  87.08 | 83.84  87.30 | 82.25  85.47 | 83.33  85.47 | 88.11  90.56 |
| CD0610 | 83.57  87.30 | 83.84  86.83 | - | 81.31  84.03 | 81.35  84.03 | 81.31  84.03 | 83.02  85.78 | 72.77  73.81 | 81.51  84.53 | 82.87  85.31 | 82.48  86.25 | 82.36  85.90 | 83.84  87.41 | 99.88  99.88 | 43.27  31.99 | 82.56  84.73 | 81.31  84.03 | 82.85  84.40 | 82.56  85.66 | 80.08  83.26 | 80.31  82.21 | 82.60  85.78 |
| CD06161 | 83.31  87.51 | 83.29  86.13 | 81.31  84.03 | - | 99.96  99.88 | 99.84  99.53 | 85.03  86.93 | 73.55  74.71 | 84.98  88.47 | 84.68  87.75 | 85.18  88.45 | 82.44  85.78 | 83.76  87.06 | 81.43  84.15 | 42.66  31.66 | 82.26  87.05 | 99.81  99.53 | 82.67  85.90 | 82.30  86.23 | 83.74  87.19 | 84.71  87.66 | 84.42  86.60 |
| CD06162 | 83.35  87.51 | 83.33  86.13 | 81.35  84.03 | **99.96**  99.88 | - | 99.81  99.53 | 85.06  86.93 | 73.51  74.59 | 85.02  88.47 | 84.71  87.75 | 85.22  88.45 | 82.48  85.78 | 83.80  87.18 | 81.47  84.15 | 42.62  31.56 | 82.30  87.05 | 99.77  99.53 | 82.71  85.90 | 82.34  86.23 | 83.78  87.19 | 84.75  87.66 | 84.38  86.48 |
| CD0617 | 83.24  87.16 | 83.22  85.78 | 81.31  84.03 | 99.84  99.53 | 99.81  99.53 | - | 84.95  86.58 | 73.40  74.25 | 84.90  88.13 | 84.60  87.40 | 85.18  88.33 | 82.36  85.55 | 83.61  86.71 | 81.43  84.15 | 42.58  31.66 | 82.19  86.81 | 99.96  100 | 82.60  85.55 | 82.22  85.88 | 83.66  86.85 | 84.63  87.31 | 84.27  86.13 |
| CD0523 | 86.81  89.73 | 85.04  88.11 | 83.02  85.78 | 85.03  86.93 | 85.06  86.93 | 84.95  86.58 | - | 73.70  75.64 | 86.34  89.52 | 87.24  90.08 | 86.81  89.15 | 84.46  88.23 | 85.82  89.39 | 83.14  85.90 | 41.64  31.45 | 86.70  89.73 | 84.95  86.58 | 85.35  88.34 | 86.23  89.61 | 82.62  86.26 | 83.93  86.03 | 84.23  87.18 |
| CD0524 | 73.90  76.80 | 73.54  75.55 | 72.77  73.81 | 73.55  74.71 | 73.51  74.59 | 73.40  74.25 | 73.70  75.64 | - | 73.65  75.00 | 73.47  75.29 | 74.60  76.31 | 72.92  74.51 | 74.24  76.25 | 72.73  73.70 | 43.13  30.59 | 73.32  75.52 | 73.36  74.25 | 74.39  76.01 | 73.75  74.80 | 72.07  73.50 | 73.38  74.31 | 73.39  75.32 |
| LS0612 | 85.72  89.64 | 83.72  87.44 | 81.51  84.53 | 84.98  88.47 | 85.02  88.47 | 84.90  88.13 | 86.34  89.52 | 73.65  75.00 | - | 84.56  89.06 | 84.75  87.19 | 85.81  88.49 | 84.50  89.19 | 81.55  84.65 | 42.38  31.60 | 84.01  88.71 | 84.87  88.13 | 83.91  87.91 | 83.86  87.66 | 85.56  88.82 | 85.22  87.78 | 83.22  86.16 |
| DY0615 | 87.36  91.48 | 85.66  88.46 | 82.87  85.31 | 84.68  87.75 | 84.71  87.75 | 84.60  87.40 | 87.24  90.08 | 73.47  75.29 | 84.56  89.06 | - | 85.96  89.03 | 83.57  88.00 | 85.74  89.51 | 82.98  85.43 | 42.22  31.77 | 89.11  91.02 | 84.60  87.40 | 84.97  87.88 | 86.74  89.96 | 82.27  86.15 | 83.12  85.68 | 85.04  88.00 |
| CD0607 | 85.57  90.55 | 85.20  89.16 | 82.48  86.25 | 85.18  88.45 | 85.22  88.45 | 85.18  88.33 | 86.81  89.15 | 74.60  76.31 | 84.75  87.19 | 85.96  89.03 | - | 84.19  88.11 | 84.54  88.46 | 82.60  86.36 | 42.22  31.45 | 86.58  89.96 | 85.18  88.33 | 83.06  86.36 | 86.93  89.26 | 82.62  87.19 | 84.01  86.96 | 84.50  86.95 |
| CD0521 | 84.34  87.88 | 88.54  91.84 | 82.36  85.90 | 82.44  85.78 | 82.48  85.78 | 82.36  85.55 | 84.46  88.23 | 72.92  74.51 | 85.81  88.49 | 83.57  88.00 | 84.19  88.11 | - | 89.04  92.31 | 82.48  86.01 | 42.87  31.66 | 83.64  87.41 | 82.36  85.55 | 83.74  87.19 | 83.29  87.30 | 81.59  85.47 | 83.06  85.35 | 86.64  90.79 |
| SN0623 | 84.58  89.86 | 89.20  92.77 | 83.84  87.41 | 83.76  87.06 | 83.80  87.18 | 83.61  86.71 | 85.82  89.39 | 74.24  76.25 | 84.50  89.19 | 85.74  89.51 | 84.54  88.46 | 89.04  92.31 | - | 83.95  87.53 | 42.69  31.12 | 84.65  89.39 | 83.61  86.71 | 85.41  88.59 | 83.80  87.88 | 81.90  86.51 | 82.71  86.16 | 89.01  92.31 |
| GY0528 | 83.68  87.41 | 83.95  86.95 | 99.88  99.88 | 81.43  84.15 | 81.47  84.15 | 81.43  84.15 | 83.14  85.90 | 72.73  73.70 | 81.55  84.65 | 82.98  85.43 | 82.60  86.36 | 82.48  86.01 | 83.95  87.53 | - | 43.27  32.10 | 82.67  84.85 | 81.43  84.15 | 82.96  84.52 | 82.67  85.78 | 80.19  83.37 | 80.43  82.33 | 82.71  85.90 |
| **MY0628** | 42.18  31.77 | 42.58  31.66 | 43.27  31.99 | 42.66  31.66 | 42.62  31.56 | 42.58  31.66 | **41.64**  31.45 | 43.13  **30.59** | 42.38  31.60 | 42.22  31.77 | 42.22  31.45 | 42.87  31.66 | 42.69  31.12 | 43.27  32.10 | - | 42.15  31.88 | 42.58  31.66 | 42.10  30.76 | 42.62  31.88 | 43.48  31.62 | 42.74  31.70 | 42.69  31.88 |
| CD0402 | 86.43  90.43 | 84.81  88.00 | 82.56  84.73 | 82.26  87.05 | 82.30  87.05 | 82.19  86.81 | 86.70  89.73 | 73.32  75.52 | 84.01  88.71 | 89.11  91.02 | 86.58  89.96 | 83.64  87.41 | 84.65  89.39 | 82.67  84.85 | 42.15  31.88 | - | 82.19  86.81 | 83.68  86.60 | 85.96  89.38 | 82.27  86.85 | 81.99  85.33 | 82.48  86.01 |
| **CD0522** | 83.24  87.16 | 83.22  85.78 | 81.31  84.03 | 99.81  99.53 | 99.77  99.53 | **99.96**  **100** | 84.95  86.58 | 73.36  74.25 | 84.87  88.13 | 84.60  87.40 | 85.18  88.33 | 82.36  85.55 | 83.61  86.71 | 81.43  84.15 | 42.58  31.66 | 82.19  86.81 | - | 82.60  85.55 | 82.22  85.88 | 83.62  86.85 | 84.59  87.31 | 84.27  86.13 |
| CDQY0608 | 84.38  87.53 | 84.75  87.08 | 82.85  84.40 | 82.67  85.90 | 82.71  85.90 | 82.60  85.55 | 85.35  88.34 | 74.39  76.01 | 83.91  87.91 | 84.97  87.88 | 83.06  86.36 | 83.74  87.19 | 85.41  88.59 | 82.96  84.52 | 42.10  30.76 | 83.68  86.60 | 82.60  85.55 | - | 83.02  85.90 | 81.51  85.00 | 83.37  84.42 | 83.62  85.91 |
| CD0614 | 85.38  89.85 | 83.84  87.30 | 82.56  85.66 | 82.30  86.23 | 82.34  86.23 | 82.22  85.88 | 86.23  89.61 | 73.75  74.80 | 83.86  87.66 | 86.74  89.96 | 86.93  89.26 | 83.29  87.30 | 83.80  87.88 | 82.67  85.78 | 42.62  31.88 | 85.96  89.38 | 82.22  85.88 | 83.02  85.90 | - | 82.15  85.45 | 84.32  86.50 | 82.94  86.60 |
| JN0608 | 82.15  87.66 | 82.25  85.47 | 80.08  83.26 | 83.74  87.19 | 83.78  87.19 | 83.66  86.85 | 82.62  86.26 | 72.07  73.50 | 85.56  88.82 | 82.27  86.15 | 82.62  87.19 | 81.59  85.47 | 81.90  86.51 | 80.19  83.37 | 43.48  31.62 | 82.27  86.85 | 83.62  86.85 | 81.51  85.00 | 82.15  85.45 | - | 84.44  88.24 | 81.16  83.49 |
| CD0525 | 83.35  86.38 | 83.33  85.47 | 80.31  82.21 | 84.71  87.66 | 84.75  87.66 | 84.63  87.31 | 83.93  86.03 | 73.38  74.31 | 85.22  87.78 | 83.12  85.68 | 84.01  86.96 | 83.06  85.35 | 82.71  86.16 | 80.43  82.33 | 42.74  31.70 | 81.99  85.33 | 84.59  87.31 | 83.37  84.42 | 84.32  86.50 | 84.44  88.24 | - | 81.98  84.42 |
| DY0528 | 83.37  87.18 | 88.11  90.56 | 82.60  85.78 | 84.42  86.60 | 84.38  86.48 | 84.27  86.13 | 84.23  87.18 | 73.39  75.32 | 83.22  86.16 | 85.04  88.00 | 84.50  86.95 | 86.64  90.79 | 89.01  92.31 | 82.71  85.90 | 42.69  31.88 | 82.48  86.01 | 84.27  86.13 | 83.62  85.91 | 82.94  86.60 | 81.16  83.49 | 81.98  84.42 | - |

*The red label represents the maximum value, and the blue label represents the minimum value. Each table cell has two items, the upper part is the nucleotide similarity, and the lower part is the amino acid similarity.

Table S3. FCoV N Gene Sequence Identity Matrix

|  | MY0622 | LS0610 | CD0610 | CD06161 | CD06162 | **CD0523** | CD0524 | LS0612 | CD0521 | SN0623 | GY0528 | MY0628 | **CD0402** | CD0522 | CDQY0608 | LS0530 | LS0606 | DY0522 | DY0523 | CD0609 | CD0615 | CD0617 | CD0525 | DY0612 | LS0522 |
| --- | --- | --- | --- | --- | --- | --- | --- | --- | --- | --- | --- | --- | --- | --- | --- | --- | --- | --- | --- | --- | --- | --- | --- | --- | --- |
| MY0622 | - | 92.68  94.71 | 91.36  91.53 | 93.65  96.03 | 92.19  93.68 | 91.09  91.53 | 94.00  95.50 | 91.98  94.44 | 93.21  93.92 | 93.30  93.92 | 92.68  94.18 | 93.65  94.18 | 93.12  94.44 | 94.62  94.97 | 93.21  94.44 | 91.80  91.01 | 92.86  93.92 | 93.21  93.65 | 93.03  95.24 | 92.15  94.18 | 91.18  91.27 | 93.03  94.71 | 92.77  94.44 | 92.50  92.86 | 93.39  94.97 |
| LS0610 | 92.68  94.71 | - | 92.33  92.86 | 94.44  95.77 | 92.46  93.16 | 90.56  91.27 | 92.86  94.97 | 94.27  95.77 | 93.56  95.24 | 93.74  93.65 | 93.21  94.97 | 92.77  93.92 | 91.98  93.39 | 93.39  94.44 | 92.06  93.39 | 91.62  91.80 | 92.68  94.71 | 93.12  94.71 | 93.56  96.03 | 93.56  95.50 | 92.06  92.86 | 93.56  95.50 | 93.83  95.24 | 93.56  94.44 | 94.53  96.03 |
| CD0610 | 91.36  91.53 | 92.33  92.86 | - | 92.33  92.06 | 90.96  91.05 | 92.42  92.06 | 91.98  91.53 | 91.89  93.12 | 92.50  92.06 | 92.77  92.86 | 92.42  91.80 | 92.24  91.27 | 93.03  94.18 | 92.42  91.27 | 93.12  94.18 | 92.15  91.01 | 91.62  92.06 | 92.33  91.27 | 91.45  93.39 | 91.45  92.06 | 93.56  93.65 | 91.62  92.86 | 92.24  93.12 | 93.83  92.86 | 92.59  93.12 |
| CD06161 | 93.65  96.03 | 94.44  95.77 | 92.33  92.06 | - | 94.27  95.24 | 90.56  91.01 | 92.77  95.50 | 93.39  95.77 | 94.44  95.77 | 93.47  95.24 | 94.09  96.03 | 93.83  96.03 | 92.15  94.18 | 94.36  96.03 | 92.24  94.18 | 92.33  91.53 | 92.68  94.97 | 93.92  95.24 | 93.30  96.56 | 93.30  94.97 | 91.98  91.80 | 92.95  96.03 | 94.27  95.50 | 93.21  93.65 | 94.53  96.03 |
| CD06162 | 92.19  93.68 | 92.46  93.16 | 90.96  91.05 | 94.27  95.24 | - | 90.53  91.32 | 91.75  93.68 | 91.84  93.95 | 92.28  93.16 | 92.89  92.89 | 92.63  93.68 | 93.25  95.26 | 92.02  93.16 | 93.33  93.95 | 92.11  93.16 | 91.14  90.26 | 91.67  92.89 | 92.98  93.95 | 93.42  96.05 | 91.75  92.37 | 91.75  92.11 | 93.16  95.53 | 92.89  94.74 | 92.54  93.68 | 93.95  93.95 |
| CD0523 | 91.09  91.53 | 90.56  91.27 | 92.42  92.06 | 90.56  91.01 | 90.53  91.32 | - | 91.45  92.33 | 91.09  92.33 | 91.89  91.80 | 91.62  91.53 | 91.16  90.72 | 91.53  91.01 | 93.12  93.39 | 91.71  90.48 | 93.21  93.39 | 91.27  89.42 | 90.92  91.27 | 90.39  90.48 | 91.01  93.12 | 90.04  89.95 | 91.89  92.06 | 90.65  92.59 | 90.65  91.53 | 92.59  92.06 | 91.27  92.33 |
| CD0524 | 94.00  95.50 | 92.86  94.97 | 91.98  91.53 | 92.77  95.50 | 91.75  93.68 | 91.45  92.33 | - | 92.42  94.71 | 92.15  93.92 | 93.39  94.18 | 92.95  94.71 | 93.30  94.18 | 93.56  94.18 | 94.09  94.97 | 93.65  94.18 | 92.33  91.80 | 92.68  94.71 | 92.68  94.18 | 93.03  95.77 | 92.59  93.65 | 92.33  92.33 | 92.68  95.24 | 93.39  94.97 | 93.74  93.65 | 93.39  95.24 |
| LS0612 | 91.98  94.44 | 94.27  95.77 | 91.89  93.12 | 93.39  95.77 | 91.84  93.95 | 91.09  92.33 | 92.42  94.71 | - | 92.68  94.97 | 94.27  96.30 | 93.56  95.50 | 93.92  95.24 | 92.33  94.71 | 93.12  94.97 | 92.42  94.71 | 91.36  91.80 | 92.86  95.24 | 93.30  95.24 | 93.74  97.09 | 93.56  94.71 | 91.62  92.86 | 93.56  96.56 | 94.09  95.77 | 92.77  94.44 | 93.56  96.03 |
| CD0521 | 93.21  93.92 | 93.56  95.24 | 92.50  92.06 | 94.44  95.77 | 92.28  93.16 | 91.89  91.80 | 92.15  93.92 | 92.68  94.97 | - | 92.42  93.65 | 92.77  94.44 | 93.47  94.44 | 92.50  93.65 | 93.65  93.92 | 92.59  93.65 | 92.06  90.48 | 92.86  93.65 | 93.74  94.44 | 92.86  95.77 | 93.03  93.92 | 91.62  92.59 | 92.68  95.24 | 93.74  94.18 | 92.50  93.39 | 93.83  95.24 |
| SN0623 | 93.30  93.92 | 93.74  93.65 | 92.77  92.86 | 93.47  95.24 | 92.89  92.89 | 91.62  91.53 | 93.39  94.18 | 94.27  96.30 | 92.42  93.65 | - | 93.47  93.92 | 94.36  94.18 | 93.74  94.71 | 93.83  93.92 | 93.83  94.71 | 91.89  91.27 | 93.47  93.92 | 93.74  94.18 | 94.00  95.24 | 92.95  93.65 | 92.06  91.80 | 93.56  94.71 | 93.12  94.71 | 92.77  92.59 | 93.83  94.44 |
| GY0528 | 92.68  94.18 | 93.21  94.97 | 92.42  91.80 | 94.09  96.03 | 92.63  93.68 | 91.16  90.72 | 92.95  94.71 | 93.56  95.50 | 92.77  94.44 | 93.47  93.92 | - | 93.47  94.71 | 92.42  92.59 | 94.09  94.97 | 92.50  92.59 | 92.24  91.27 | 93.21  94.71 | 93.56  95.50 | 94.09  96.30 | 92.50  94.71 | 91.98  91.53 | 92.68  95.77 | 93.39  95.50 | 92.95  93.65 | 94.18  95.24 |
| MY0628 | 93.65  94.18 | 92.77  93.92 | 92.24  91.27 | 93.83  96.03 | 93.25  95.26 | 91.53  91.01 | 93.30  94.18 | 93.92  95.24 | 93.47  94.44 | 94.36  94.18 | 93.47  94.71 | - | 93.47  93.65 | 95.06  95.50 | 93.56  93.65 | 92.15  91.01 | 93.21  93.39 | 94.97  94.97 | 94.18  96.56 | 92.42  93.12 | 92.24  92.33 | 94.18  96.03 | 94.62  95.50 | 93.74  94.44 | 93.83  94.71 |
| CD0402 | 93.12  94.44 | 91.98  93.39 | 93.03  94.18 | 92.15  94.18 | 92.02  93.16 | 93.12  93.39 | 93.56  94.18 | 92.33  94.71 | 92.50  93.65 | 93.74  94.71 | 92.42  92.59 | 93.47  93.65 | - | 93.47  92.86 | 99.91  100 | 92.15  92.59 | 92.77  93.39 | 92.68  92.86 | 92.42  94.97 | 92.15  93.12 | 92.24  93.65 | 92.59  94.97 | 93.12  94.44 | 93.47  93.39 | 93.56  94.71 |
| CD0522 | 94.62  94.97 | 93.39  94.44 | 92.42  91.27 | 94.36  96.03 | 93.33  93.95 | 91.71  90.48 | 94.09  94.97 | 93.12  94.97 | 93.65  93.92 | 93.83  93.92 | 94.09  94.97 | 95.06  95.50 | 93.47  92.86 | - | 93.56  92.86 | 91.98  91.27 | 94.00  94.44 | 94.00  94.44 | 93.92  96.03 | 92.86  94.18 | 92.15  91.80 | 93.74  95.50 | 94.18  95.50 | 94.18  94.18 | 94.00  94.71 |
| **CDQY0608** | 93.21  94.44 | 92.06  93.39 | 93.12  94.18 | 92.24  94.18 | 92.11  93.16 | 93.21  93.39 | 93.65  94.18 | 92.42  94.71 | 92.59  93.65 | 93.83  94.71 | 92.50  92.59 | 93.56  93.65 | **99.91**  **100** | 93.56  92.86 | - | 92.24  92.59 | 92.86  93.39 | 92.77  92.86 | 92.50  94.97 | 92.24  93.12 | 92.33  93.65 | 92.68  94.97 | 93.21  94.44 | 93.56  93.39 | 93.47  94.71 |
| **LS0530** | 91.80  91.01 | 91.62  91.80 | 92.15  91.01 | 92.33  91.53 | 91.14  90.26 | 91.27  **89.42** | 92.33  91.80 | 91.36  91.80 | 92.06  90.48 | 91.89  91.27 | 92.24  91.27 | 92.15  91.01 | 92.15  92.59 | 91.98  91.27 | 92.24  92.59 | - | 93.56  93.12 | 91.98  90.48 | 91.62  93.39 | 90.92  91.27 | 92.15  91.01 | 91.09  92.86 | 91.98  93.12 | 92.59  91.53 | 92.42  92.59 |
| LS0606 | 92.86  93.92 | 92.68  94.71 | 91.62  92.06 | 92.68  94.97 | 91.67  92.89 | 90.92  91.27 | 92.68  94.71 | 92.86  95.24 | 92.86  93.65 | 93.47  93.92 | 93.21  94.71 | 93.21  93.39 | 92.77  93.39 | 94.00  94.44 | 92.86  93.39 | 93.56  93.12 | - | 93.39  94.18 | 92.86  95.50 | 92.68  94.97 | 91.71  92.86 | 92.68  94.97 | 92.15  94.71 | 92.24  93.39 | 93.12  95.50 |
| DY0522 | 93.21  93.65 | 93.12  94.71 | 92.33  91.27 | 93.92  95.24 | 92.98  93.95 | 90.39  90.48 | 92.68  94.18 | 93.30  95.24 | 93.74  94.44 | 93.74  94.18 | 93.56  95.50 | 94.97  94.97 | 92.68  92.86 | 94.00  94.44 | 92.77  92.86 | 91.98  90.48 | 93.39  94.18 | - | 93.56  95.77 | 92.68  94.71 | 91.89  91.01 | 93.47  95.24 | 93.03  94.97 | 92.86  93.12 | 94.80  94.97 |
| DY0523 | 93.03  95.24 | 93.56  96.03 | 91.45  93.39 | 93.30  96.56 | 93.42  96.05 | 91.01  93.12 | 93.03  95.77 | 93.74  97.09 | 92.86  95.77 | 94.00  95.24 | 94.09  96.30 | 94.18  96.56 | 92.42  94.97 | 93.92  96.03 | 92.50  94.97 | 91.62  93.39 | 92.86  95.50 | 93.56  95.77 | - | 93.12  95.77 | 91.89  93.65 | 98.59  99.47 | 94.18  97.88 | 93.12  95.77 | 94.44  96.56 |
| **CD0609** | 92.15  94.18 | 93.56  95.50 | 91.45  92.06 | 93.30  94.97 | 91.75  92.37 | **90.04**  89.95 | 92.59  93.65 | 93.56  94.71 | 93.03  93.92 | 92.95  93.65 | 92.50  94.71 | 92.42  93.12 | 92.15  93.12 | 92.86  94.18 | 92.24  93.12 | 90.92  91.27 | 92.68  94.97 | 92.68  94.71 | 93.12  95.77 | - | 91.09  90.48 | 92.86  95.24 | 93.56  94.97 | 92.59  92.59 | 93.03  95.24 |
| CD0615 | 91.18  91.27 | 92.06  92.86 | 93.56  93.65 | 91.98  91.80 | 91.75  92.11 | 91.89  92.06 | 92.33  92.33 | 91.62  92.86 | 91.62  92.59 | 92.06  91.80 | 91.98  91.53 | 92.24  92.33 | 92.24  93.65 | 92.15  91.80 | 92.33  93.65 | 92.15  91.01 | 91.71  92.86 | 91.89  91.01 | 91.89  93.65 | 91.09  90.48 | - | 91.53  93.12 | 91.98  93.12 | 96.65  96.30 | 92.33  92.59 |
| CD0617 | 93.03  94.71 | 93.56  95.50 | 91.62  92.86 | 92.95  96.03 | 93.16  95.53 | 90.65  92.59 | 92.68  95.24 | 93.56  96.56 | 92.68  95.24 | 93.56  94.71 | 92.68  95.77 | 94.18  96.03 | 92.59  94.97 | 93.74  95.50 | 92.68  94.97 | 91.09  92.86 | 92.68  94.97 | 93.47  95.24 | 98.59  99.47 | 92.86  95.24 | 91.53  93.12 | - | 94.18  97.35 | 93.12  95.24 | 94.27  96.03 |
| CD0525 | 92.77  94.44 | 93.83  95.24 | 92.24  93.12 | 94.27  95.50 | 92.89  94.74 | 90.65  91.53 | 93.39  94.97 | 94.09  95.77 | 93.74  94.18 | 93.12  94.71 | 93.39  95.50 | 94.62  95.50 | 93.12  94.44 | 94.18  95.50 | 93.21  94.44 | 91.98  93.12 | 92.15  94.71 | 93.03  94.97 | 94.18  97.88 | 93.56  94.97 | 91.98  93.12 | 94.18  97.35 | - | 95.15  96.30 | 94.09  95.50 |
| DY0612 | 92.50  92.86 | 93.56  94.44 | 93.83  92.86 | 93.21  93.65 | 92.54  93.68 | 92.59  92.06 | 93.74  93.65 | 92.77  94.44 | 92.50  93.39 | 92.77  92.59 | 92.95  93.65 | 93.74  94.44 | 93.47  93.39 | 94.18  94.18 | 93.56  93.39 | 92.59  91.53 | 92.24  93.39 | 92.86  93.12 | 93.12  95.77 | 92.59  92.59 | 96.65  96.30 | 93.12  95.24 | 95.15  96.30 | - | 93.65  93.92 |
| LS0522 | 93.39  94.97 | 94.53  96.03 | 92.59  93.12 | 94.53  96.03 | 93.95  93.95 | 91.27  92.33 | 93.39  95.24 | 93.56  96.03 | 93.83  95.24 | 93.83  94.44 | 94.18  95.24 | 93.83  94.71 | 93.56  94.71 | 94.00  94.71 | 93.47  94.71 | 92.42  92.59 | 93.12  95.50 | 94.80  94.97 | 94.44  96.56 | 93.03  95.24 | 92.33  92.59 | 94.27  96.03 | 94.09  95.50 | 93.65  93.92 | - |

Table S4. FCoV M Gene Sequence Identity Matrix

|  | MY0622 | LS0610 | **CD0610** | CD06161 | CD06162 | **CD0523** | **CD0524** | LS0612 | DY0615 | CD0607 | CD0521 | SN0623 | **GY0528** | MY0628 | CDQY68 | LS0526 | LS0530 | LS0606 | DY0522 | DY0523 | CD0614 | CDJN68 | CD0617 | CD0525 | DY0528 | DY0612 |
| --- | --- | --- | --- | --- | --- | --- | --- | --- | --- | --- | --- | --- | --- | --- | --- | --- | --- | --- | --- | --- | --- | --- | --- | --- | --- | --- |
| MY0622 | - | 90.31  93.21 | 91.45  95.47 | 90.94  94.34 | 90.82  94.34 | 90.44  93.58 | 81.20  83.83 | 91.45  95.09 | 89.43  93.21 | 91.19  94.34 | 92.70  96.23 | 91.57  95.09 | 80.83  83.83 | 86.84  89.85 | 81.08  83.83 | 84.53  87.92 | 80.95  83.46 | 91.07  93.58 | 93.08  95.47 | 83.02  86.79 | 91.45  94.72 | 90.31  93.58 | 92.33  93.96 | 92.83  95.09 | 91.70  95.09 | 90.82  93.21 |
| LS0610 | 90.31  93.21 | - | 91.57  93.58 | 92.30  94.32 | 92.42  94.32 | 91.67  93.94 | 82.77  84.91 | 92.30  93.94 | 90.53  92.80 | 92.42  94.32 | 90.57  94.34 | 92.17  94.32 | 82.64  84.91 | 89.06  90.19 | 82.89  84.91 | 85.91  88.30 | 82.77  84.53 | 91.04  93.94 | 91.32  92.83 | 84.97  87.50 | 91.79  93.94 | 91.79  93.94 | 91.95  93.58 | 91.95  93.58 | 91.70  94.72 | 92.30  95.45 |
| CD0610 | 91.45  95.47 | 91.57  93.58 | - | 91.92  94.32 | 92.05  94.32 | 90.69  92.83 | 80.45  82.71 | 91.41  93.56 | 91.04  92.80 | 90.82  92.83 | 91.32  93.96 | 91.70  93.96 | 80.58  82.71 | 87.09  88.72 | 80.58  82.71 | 84.28  87.17 | 80.45  82.33 | 90.82  92.83 | 92.08  95.09 | 82.89  86.42 | 91.70  93.58 | 90.82  92.83 | 91.45  92.83 | 91.95  93.58 | 91.95  94.34 | 90.31  91.70 |
| CD06161 | 90.94  94.34 | 92.30  94.32 | 91.92  94.32 | - | 99.87  100 | 92.80  95.08 | 83.14  86.79 | 92.90  96.20 | 93.03  95.82 | 92.55  95.08 | 92.70  95.47 | 92.68  95.45 | 82.77  86.79 | 88.81  92.08 | 83.02  86.79 | 84.53  89.06 | 82.89  86.42 | 91.79  95.08 | 91.07  93.96 | 83.84  87.12 | 92.80  95.83 | 92.68  95.08 | 92.83  93.96 | 92.45  94.34 | 91.95  95.47 | 92.80  94.70 |
| CD06162 | 90.82  94.34 | 92.42  94.32 | 92.05  94.32 | **99.87**  **100** | - | 92.68  95.08 | 83.02  86.79 | 92.78  96.20 | 92.90  95.82 | 92.68  95.08 | 92.58  95.47 | 92.80  95.45 | 82.89  86.79 | 88.93  92.08 | 83.14  86.79 | 84.40  89.06 | 83.02  86.42 | 91.92  95.08 | 90.94  93.96 | 83.71  87.12 | 92.93  95.83 | 92.80  95.08 | 92.96  93.96 | 92.33  94.34 | 91.82  95.47 | 92.68  94.70 |
| CD0523 | 90.44  93.58 | 91.67  93.94 | 90.69  92.83 | 92.80  95.08 | 92.68  95.08 | - | 83.14  84.91 | 92.93  95.08 | 92.93  95.45 | 92.68  93.94 | 92.83  95.47 | 93.31  95.45 | 82.77  84.91 | 88.30  90.94 | 83.02  84.91 | 84.78  87.17 | 82.89  84.53 | 92.05  95.08 | 91.19  93.58 | 84.60  88.26 | 93.31  95.83 | 99.87  100 | 92.08  93.96 | 92.20  94.34 | 92.33  94.34 | 93.81  95.45 |
| CD0524 | 81.20  83.83 | 82.77  84.91 | 80.45  82.71 | 83.14  86.79 | 83.02  86.79 | 83.14  84.91 | - | 82.52  84.91 | 81.76  84.53 | 82.52  84.91 | 81.83  84.96 | 81.13  84.15 | 99.49  100 | 90.15  92.05 | 99.87  100 | 92.61  91.73 | 99.75  99.62 | 80.38  84.53 | 80.20  83.08 | 87.88  89.39 | 81.01  84.53 | 83.02  84.91 | 81.83  84.21 | 82.33  82.71 | 82.08  84.59 | 82.14  85.66 |
| LS0612 | 91.45  95.09 | 92.30  93.94 | 91.41  93.56 | 92.90  96.20 | 92.78  96.20 | 92.93  95.08 | 82.52  84.91 | - | 92.40  95.06 | 93.31  95.08 | 92.33  95.47 | 93.31  95.83 | 82.39  84.91 | 89.43  90.94 | 82.39  84.91 | 85.03  87.55 | 82.26  84.53 | 92.55  95.08 | 91.45  93.58 | 83.71  87.50 | 93.31  96.21 | 92.80  95.08 | 92.45  94.34 | 92.08  94.34 | 91.95  94.72 | 92.55  95.83 |
| DY0615 | 89.43  93.21 | 90.53  92.80 | 91.04  92.80 | 93.03  95.82 | 92.90  95.82 | 92.93  95.45 | 81.76  84.53 | 92.40  95.06 | - | 92.42  93.94 | 90.94  94.34 | 92.05  94.70 | 81.64  84.53 | 87.67  90.57 | 81.64  84.53 | 83.65  86.79 | 81.51  84.15 | 90.40  93.94 | 89.69  92.83 | 82.83  87.12 | 92.17  95.08 | 92.80  95.45 | 90.69  92.45 | 90.44  93.21 | 91.57  93.96 | 91.92  93.94 |
| CD0607 | 91.19  94.34 | 92.42  94.32 | 90.82  92.83 | 92.55  95.08 | 92.68  95.08 | 92.68  93.94 | 82.52  84.91 | 93.31  95.08 | 92.42  93.94 | - | 93.46  95.47 | 95.08  95.45 | 82.39  84.91 | 88.43  90.94 | 82.64  84.91 | 85.91  88.30 | 82.52  84.53 | 92.55  93.94 | 91.57  93.58 | 83.46  86.36 | 94.95  95.08 | 92.80  93.94 | 92.83  93.58 | 93.84  95.09 | 92.96  95.47 | 92.93  94.32 |
| CD0521 | 92.70  96.23 | 90.57  94.34 | 91.32  93.96 | 92.70  95.47 | 92.58  95.47 | 92.83  95.47 | 81.83  84.96 | 92.33  95.47 | 90.94  94.34 | 93.46  95.47 | - | 92.83  95.85 | 81.45  84.96 | 87.34  90.98 | 81.70  84.96 | 85.16  89.06 | 81.58  84.59 | 92.33  95.09 | 93.58  95.09 | 83.90  88.30 | 93.21  96.23 | 92.70  95.47 | 95.22  97.36 | 93.84  95.85 | 92.08  96.23 | 92.58  94.34 |
| SN0623 | 91.57  95.09 | 92.17  94.32 | 91.70  93.96 | 92.68  95.45 | 92.80  95.45 | 93.31  95.45 | 81.13  84.15 | 93.31  95.83 | 92.05  94.70 | 95.08  95.45 | 92.83  95.85 | - | 81.01  84.15 | 87.42  90.19 | 81.26  84.15 | 85.53  88.68 | 81.13  83.77 | 92.17  94.70 | 92.58  94.34 | 83.21  87.50 | 99.37  99.62 | 93.43  95.45 | 92.70  93.96 | 92.58  94.72 | 93.08  95.85 | 91.79  93.94 |
| GY0528 | 80.83  83.83 | 82.64  84.91 | 80.58  82.71 | 82.77  86.79 | 82.89  86.79 | 82.77  84.91 | 99.49  100 | 82.39  84.91 | 81.64  84.53 | 82.39  84.91 | 81.45  84.96 | 81.01  84.15 | - | 90.28  92.05 | 99.62  100 | 92.61  91.73 | 99.49  99.62 | 80.25  84.53 | 79.82  83.08 | 87.75  89.39 | 80.88  84.53 | 82.89  84.91 | 81.70  84.21 | 81.95  82.71 | 81.95  84.59 | 81.76  85.66 |
| MY0628 | 86.84  89.85 | 89.06  90.19 | 87.09  88.72 | 88.81  92.08 | 88.93  92.08 | 88.30  90.94 | 90.15  92.05 | 89.43  90.94 | 87.67  90.57 | 88.43  90.94 | 87.34  90.98 | 87.42  90.19 | 90.28  92.05 | - | 90.28  92.05 | 83.21  84.21 | 90.15  91.67 | 86.92  90.19 | 86.34  88.35 | 86.99  88.26 | 87.30  90.57 | 88.43  90.94 | 87.34  89.47 | 88.10  89.47 | 87.72  90.60 | 87.80  90.94 |
| **CDQY68** | 81.08  83.83 | 82.89  84.91 | 80.58  82.71 | 83.02  86.79 | 83.14  86.79 | 83.02  84.91 | **99.87**  **100** | 82.39  84.91 | 81.64  84.53 | 82.64  84.91 | 81.70  84.96 | 81.26  84.15 | 99.62  100 | 90.28  92.05 | - | 92.48  91.73 | 99.87  99.62 | 80.50  84.53 | 80.08  83.08 | 87.75  89.39 | 81.13  84.53 | 83.14  84.91 | 81.95  84.21 | 82.21  82.71 | 81.95  84.59 | 82.01  85.66 |
| LS0526 | 84.53  87.92 | 85.91  88.30 | 84.28  87.17 | 84.53  89.06 | 84.40  89.06 | 84.78  87.17 | 92.61  91.73 | 85.03  87.55 | 83.65  86.79 | 85.91  88.30 | 85.16  89.06 | 85.53  88.68 | 92.61  91.73 | 83.21  84.21 | 92.48  91.73 | - | 92.36  91.35 | 83.27  87.55 | 84.65  88.30 | 86.54  86.79 | 85.16  88.30 | 84.65  87.17 | 85.28  88.30 | 87.67  89.43 | 89.06  92.45 | 83.65  87.92 |
| **LS0530** | 80.95  83.46 | 82.77  84.53 | 80.45  **82.33** | 82.89  86.42 | 83.02  86.42 | 82.89  84.53 | 99.75  99.62 | 82.26  84.53 | 81.51  84.15 | 82.52  84.53 | 81.58  84.59 | 81.13  83.77 | 99.49  99.62 | 90.15  91.67 | 99.87  99.62 | 92.36  91.35 | - | 80.38  84.15 | 79.95  82.71 | 87.63  89.02 | 81.01  84.15 | 83.02  84.53 | 81.83  83.83 | 82.08  82.33 | 81.83  84.21 | 81.89  85.28 |
| LS0606 | 91.07  93.58 | 91.04  93.94 | 90.82  92.83 | 91.79  95.08 | 91.92  95.08 | 92.05  95.08 | 80.38  84.53 | 92.55  95.08 | 90.40  93.94 | 92.55  93.94 | 92.33  95.09 | 92.17  94.70 | 80.25  84.53 | 86.92  90.19 | 80.50  84.53 | 83.27  87.55 | 80.38  84.15 | - | 90.19  92.83 | 83.84  89.02 | 92.80  95.08 | 92.17  95.08 | 92.20  93.96 | 91.82  94.34 | 91.07  93.96 | 93.43  94.70 |
| **DY0522** | 93.08  95.47 | 91.32  92.83 | 92.08  95.09 | 91.07  93.96 | 90.94  93.96 | 91.19  93.58 | 80.20  83.08 | 91.45  93.58 | 89.69  92.83 | 91.57  93.58 | 93.58  95.09 | 92.58  94.34 | **79.82**  83.08 | 86.34  88.35 | 80.08  83.08 | 84.65  88.30 | 79.95  82.71 | 90.19  92.83 | - | 83.40  86.04 | 92.45  93.96 | 91.07  93.58 | 92.33  92.45 | 93.96  95.47 | 91.95  94.72 | 90.06  92.08 |
| DY0523 | 83.02  86.79 | 84.97  87.50 | 82.89  86.42 | 83.84  87.12 | 83.71  87.12 | 84.60  88.26 | 87.88  89.39 | 83.71  87.50 | 82.83  87.12 | 83.46  86.36 | 83.90  88.30 | 83.21  87.50 | 87.75  89.39 | 86.99  88.26 | 87.75  89.39 | 86.54  86.79 | 87.63  89.02 | 83.84  89.02 | 83.40  86.04 | - | 83.21  87.88 | 84.47  88.26 | 83.52  87.17 | 83.90  86.42 | 83.77  86.42 | 83.08  86.74 |
| CD0614 | 91.45  94.72 | 91.79  93.94 | 91.70  93.58 | 92.80  95.83 | 92.93  95.83 | 93.31  95.83 | 81.01  84.53 | 93.31  96.21 | 92.17  95.08 | 94.95  95.08 | 93.21  96.23 | 99.37  99.62 | 80.88  84.53 | 87.30  90.57 | 81.13  84.53 | 85.16  88.30 | 81.01  84.15 | 92.80  95.08 | 92.45  93.96 | 83.21  87.88 | - | 93.43  95.83 | 92.58  94.34 | 92.45  94.34 | 92.96  95.47 | 92.42  94.32 |
| **CDJN68** | 90.31  93.58 | 91.79  93.94 | 90.82  92.83 | 92.68  95.08 | 92.80  95.08 | **99.87**  **100** | 83.02  84.91 | 92.80  95.08 | 92.80  95.45 | 92.80  93.94 | 92.70  95.47 | 93.43  95.45 | 82.89  84.91 | 88.43  90.94 | 83.14  84.91 | 84.65  87.17 | 83.02  84.53 | 92.17  95.08 | 91.07  93.58 | 84.47  88.26 | 93.43  95.83 | - | 92.20  93.96 | 92.08  94.34 | 92.20  94.34 | 93.69  95.45 |
| CD0617 | 92.33  93.96 | 91.95  93.58 | 91.45  92.83 | 92.83  93.96 | 92.96  93.96 | 92.08  93.96 | 81.83  84.21 | 92.45  94.34 | 90.69  92.45 | 92.83  93.58 | 95.22  97.36 | 92.70  93.96 | 81.70  84.21 | 87.34  89.47 | 81.95  84.21 | 85.28  88.30 | 81.83  83.83 | 92.20  93.96 | 92.33  92.45 | 83.52  87.17 | 92.58  94.34 | 92.20  93.96 | - | 93.96  93.96 | 92.70  94.72 | 91.95  94.34 |
| CD0525 | 92.83  95.09 | 91.95  93.58 | 91.95  93.58 | 92.45  94.34 | 92.33  94.34 | 92.20  94.34 | 82.33  82.71 | 92.08  94.34 | 90.44  93.21 | 93.84  95.09 | 93.84  95.85 | 92.58  94.72 | 81.95  82.71 | 88.10  89.47 | 82.21  82.71 | 87.67  89.43 | 82.08  82.33 | 91.82  94.34 | 93.96  95.47 | 83.90  86.42 | 92.45  94.34 | 92.08  94.34 | 93.96  93.96 | - | 94.72  96.60 | 91.45  93.58 |
| DY0528 | 91.70  95.09 | 91.70  94.72 | 91.95  94.34 | 91.95  95.47 | 91.82  95.47 | 92.33  94.34 | 82.08  84.59 | 91.95  94.72 | 91.57  93.96 | 92.96  95.47 | 92.08  96.23 | 93.08  95.85 | 81.95  84.59 | 87.72  90.60 | 81.95  84.59 | 89.06  92.45 | 81.83  84.21 | 91.07  93.96 | 91.95  94.72 | 83.77  86.42 | 92.96  95.47 | 92.20  94.34 | 92.70  94.72 | 94.72  96.60 | - | 91.19  94.34 |
| DY0612 | 90.82  93.21 | 92.30  95.45 | 90.31  91.70 | 92.80  94.70 | 92.68  94.70 | 93.81  95.45 | 82.14  85.66 | 92.55  95.83 | 91.92  93.94 | 92.93  94.32 | 92.58  94.34 | 91.79  93.94 | 81.76  85.66 | 87.80  90.94 | 82.01  85.66 | 83.65  87.92 | 81.89  85.28 | 93.43  94.70 | 90.06  92.08 | 83.08  86.74 | 92.42  94.32 | 93.69  95.45 | 91.95  94.34 | 91.45  93.58 | 91.19  94.34 | - |

Table S5. CCoV S1 Gene Sequence Identity Matrix

|  | **NC0520** | **DZ0623** | GY0529 | GY0608 | GY0609 | NC0521 | NC0604 | CD0529 | CD0605 | SN0623 |
| --- | --- | --- | --- | --- | --- | --- | --- | --- | --- | --- |
| NC0520 | - | 43.08/32.50 | 42.92/32.25 | 42.79/32.00 | 42.79/32.00 | 42.75/31.75 | 38.29/27.65 | 42.62/32.12 | 43.21/32.25 | 42.83/32.12 |
| DZ0623 | 43.08/32.50 | - | 97.55/98.78 | 98.64/98.78 | 98.64/98.78 | 95.70/95.38 | 69.07/67.37 | 98.19/98.91 | 96.92/97.96 | 97.92/98.51 |
| GY0529 | 42.92/32.25 | 97.55/98.78 | - | 97.42/98.51 | 97.42/98.51 | 95.47/95.11 | 69.86/67.89 | 97.28/98.91 | 96.74/97.69 | 97.78/98.64 |
| **GY0608** | 42.79/32.00 | **98.64**/98.78 | 97.42/98.51 | - | 100./100 | 95.02/94.97 | 69.24/67.37 | 97.83/98.64 | 96.38/97.55 | 98.14/98.51 |
| **GY0609** | 42.79/32.00 | **98.64**/98.78 | 97.42/98.51 | **100/100** | - | 95.02/94.97 | 69.24/67.37 | 97.83/98.64 | 96.38/97.55 | 98.14/98.51 |
| NC0521 | 42.75/31.75 | 95.70/95.38 | 95.47/95.11 | 95.02/94.97 | 95.02/94.97 | - | 68.76/65.79 | 95.74/95.38 | 95.61/94.16 | 95.20/94.70 |
| **NC0604** | **38.29/27.65** | 69.07/67.37 | 69.86/67.89 | 69.24/67.37 | 69.24/67.37 | 68.76/65.79 | - | 68.98/67.37 | 69.29/67.37 | 69.55/67.63 |
| CD0529 | 42.62/32.12 | 98.19/**98.91** | 97.28/98.91 | 97.83/98.64 | 97.83/98.64 | 95.74/95.38 | 68.98/67.37 | - | 96.74/97.69 | 97.10/98.37 |
| CD0605 | 43.21/32.25 | 96.92/97.96 | 96.74/97.69 | 96.38/97.55 | 96.38/97.55 | 95.61/94.16 | 69.29/67.37 | 96.74/97.69 | - | 97.06/98.10 |
| SN0623 | 42.83/32.12 | 97.92/98.51 | 97.78/98.64 | 98.14/98.51 | 98.14/98.51 | 95.20/94.70 | 69.55/67.63 | 97.10/98.37 | 97.06/98.10 | - |

Table S6. CCoV N Gene Sequence Identity Matrix

|  | **NC0520** | LS0611 | DZ0623 | **GY0529** | **GY0608** | GY0609 | NC0521 | NC0604 | CD0529 | CD0605 | SN0628 | SN0623 |
| --- | --- | --- | --- | --- | --- | --- | --- | --- | --- | --- | --- | --- |
| NC0520 | - | 93.83/95.24 | 77.63/77.81 | 77.55/78.07 | 77.72/78.07 | 77.63/78.07 | 77.11/76.76 | 77.63/78.07 | 77.46/78.07 | 77.20/77.81 | 79.62/78.22 | 77.37/78.07 |
| LS0611 | 93.83/95.24 | - | 77.46/78.07 | 77.63/78.33 | 77.81/78.33 | 77.72/78.33 | 77.20/77.02 | 77.89/78.33 | 77.55/78.33 | 77.46/78.07 | 79.35/77.69 | 77.63/78.33 |
| DZ0623 | 77.63/77.81 | 77.46/78.07 | - | 99.56/99.74 | 99.56/99.74 | 99.65/99.74 | 98.00/97.65 | 99.48/99.74 | 99.13/99.74 | 98.78/99.48 | 88.34/90.34 | 99.22/99.74 |
| GY0529 | 77.55/78.07 | 77.63/78.33 | 99.56/99.74 | - | 99.83/100 | 99.91/100 | 98.26/97.91 | 99.74/100 | 99.39/100 | 99.04/99.74 | 88.42/90.60 | 99.48/100 |
| GY0608 | 77.72/78.07 | 77.81/78.33 | 99.56/99.74 | 99.83/100 | - | 99.91/100 | 98.43/97.91 | 99.91/100 | 99.56/100 | 99.22/99.74 | 88.60/90.60 | 99.65/100 |
| **GY0609** | 77.63/78.07 | 77.72/78.33 | 99.65/99.74 | **99.91/100** | **99.91/100** | - | 98.35/97.91 | 99.83/100 | 99.48/100 | 99.13/99.74 | 88.51/90.60 | 99.56/100 |
| **NC0521** | **77.11/76.76** | 77.20/77.02 | 98.00/97.65 | 98.26/97.91 | 98.43/97.91 | 98.35/97.91 | - | 98.35/97.91 | 98.00/97.91 | 98.17/97.65 | 87.99/89.30 | 98.43/97.91 |
| **NC0604** | 77.63/78.07 | 77.89/78.33 | 99.48/99.74 | 99.74/100 | **99.91/100** | 99.83/100 | 98.35/97.91 | - | 99.48/100 | 99.13/99.74 | 88.51/90.60 | 99.56/100 |
| CD0529 | 77.46/78.07 | 77.55/78.33 | 99.13/99.74 | 99.39/100 | 99.56/100 | 99.48/100 | 98.00/97.91 | 99.48/100 | - | 99.22/99.74 | 88.42/90.60 | 99.22/100 |
| CD0605 | 77.20/77.81 | 77.46/78.07 | 98.78/99.48 | 99.04/99.74 | 99.22/99.74 | 99.13/99.74 | 98.17/97.65 | 99.13/99.74 | 99.22/99.74 | - | 88.42/90.34 | 99.22/99.74 |
| SN0628 | 79.62/78.22 | 79.35/77.69 | 88.34/90.34 | 88.42/90.60 | 88.60/90.60 | 88.51/90.60 | 87.99/89.30 | 88.51/90.60 | 88.42/90.60 | 88.42/90.34 | - | 88.42/90.60 |
| SN0623 | 77.37/78.07 | 77.63/78.33 | 99.22/99.74 | 99.48/100 | 99.65/100 | 99.56/100 | 98.43/97.91 | 99.56/100 | 99.22/100 | 99.22/99.74 | 88.42/90.60 | - |

Table S7. CCoV M Gene Sequence Identity Matrix

|  | **NC0520** | LS0611 | DZ0623 | GY0529 | GY0608 | GY0609 | NC0521 | NC0604 | **CD0529** | CD0605 | SN0628 | SN0623 |
| --- | --- | --- | --- | --- | --- | --- | --- | --- | --- | --- | --- | --- |
| NC0520 | - | 91.57/93.58 | 80.88/84.53 | 81.64/84.53 | 81.38/84.53 | 81.51/84.15 | 80.88/83.02 | 83.52/86.42 | 81.38/84.53 | 81.51/84.53 | 82.89/87.17 | 81.76/84.91 |
| LS0611 | 91.57/93.58 | - | 81.64/84.53 | 82.01/84.53 | 81.76/84.53 | 81.89/84.15 | 81.13/83.02 | 84.03/86.42 | 82.14/84.53 | 82.26/84.53 | 83.52/86.79 | 82.26/84.91 |
| **DZ0623** | **80.88/**84.53 | 81.64/84.53 | - | 97.85/99.24 | 98.36/99.62 | 98.23/99.24 | 96.97/96.21 | 96.09/97.73 | 98.61/99.62 | 98.36/99.24 | 87.25/89.77 | 97.98/99.24 |
| GY0529 | 81.64/84.53 | 82.01/84.53 | 97.85/99.24 | - | 99.24/99.62 | 98.86/99.24 | 97.47/96.21 | 96.84/97.73 | 98.36/99.62 | 98.86/100 | 86.87/89.39 | 99.12/99.62 |
| GY0608 | 81.38/84.53 | 81.76/84.53 | 98.36/99.62 | 99.24/99.62 | - | 99.37/99.62 | 97.98/96.59 | 97.35/98.11 | 99.12/100 | 98.61/99.62 | 87.63/89.77 | 99.37/99.62 |
| GY0609 | 81.51/84.15 | 81.89/84.15 | 98.23/99.24 | 98.86/99.24 | 99.37/99.62 | - | 97.85/96.21 | 97.22/97.73 | 98.74/99.62 | 98.48/99.24 | 87.63/89.39 | 98.99/99.24 |
| **NC0521** | **80.88/83.02** | 81.13/83.02 | 96.97/96.21 | 97.47/96.21 | 97.98/96.59 | 97.85/96.21 | - | 95.58/94.70 | 97.60/96.59 | 97.35/96.21 | 86.74/89.02 | 97.60/96.21 |
| NC0604 | 83.52/86.42 | 84.03/86.42 | 96.09/97.73 | 96.84/97.73 | 97.35/98.11 | 97.22/97.73 | 95.58/94.70 | - | 96.72/98.11 | 96.21/97.73 | 87.25/90.15 | 96.97/97.73 |
| CD0529 | 81.38/84.53 | 82.14/84.53 | 98.61/99.62 | 98.36/99.62 | 99.12/**100** | 98.74/99.62 | 97.60/96.59 | 96.72/98.11 | - | 99.49/99.62 | 87.50/89.77 | 98.99/99.62 |
| **CD0605** | 81.51/84.53 | 82.26/84.53 | 98.36/99.24 | 98.86/**100** | 98.61/99.62 | 98.48/99.24 | 97.35/96.21 | 96.21/97.73 | **99.49/**99.62 | - | 86.99/89.39 | 98.99/99.62 |
| SN0628 | 82.89/87.17 | 83.52/86.79 | 87.25/89.77 | 86.87/89.39 | 87.63/89.77 | 87.63/89.39 | 86.74/89.02 | 87.25/90.15 | 87.50/89.77 | 86.99/89.39 | - | 87.37/89.39 |
| SN0623 | 81.76/84.91 | 82.26/84.91 | 97.98/99.24 | 99.12/99.62 | 99.37/99.62 | 98.99/99.24 | 97.60/96.21 | 96.97/97.73 | 98.99/99.62 | 98.99/99.62 | 87.37/89.39 | - |

Table S8. Genetic diversity of FCoV structural gene in different regions

| **Gene** | **Region** | **Sample size** | **Haplotypes** | **Haplotype diversity** | **Nucleotide diversity** |
| --- | --- | --- | --- | --- | --- |
| S1 | China | 41 | 33 | 0.972 | 0.19 |
|  | China Sichuan | 28 | 22 | 0.958 | 0.171 |
|  | China Others | 13 | 12 | 0.987 | 0.265 |
|  | Belgium | 11 | 5 | 0.709 | 0.3 |
|  | Japan | 6 | 6 | 1 | 0.328 |
|  | Netherlands | 24 | 24 | 1 | 0.131 |
|  | United Kingdom | 7 | 7 | 1 | 0.053 |
|  | United States | 23 | 20 | 0.984 | 0.29 |
|  | Europe | 45 | 37 | 0.971 | 0.137 |
|  | Americas | 23 | 20 | 0.984 | 0.29 |
|  | Asia | 47 | 39 | 0.979 | 0.218 |
|  | All | 115 | 93 | 0.99 | 0.212 |
| N | China | 77 | 66 | 0.995 | 0.059 |
|  | China Sichuan | 34 | 25 | 0.975 | 0.063 |
|  | China Others | 43 | 41 | 0.998 | 0.048 |
|  | Belgium | 11 | 2 | 0.182 | 0.017 |
|  | Japan | 6 | 6 | 1 | 0.082 |
|  | Netherlands | 24 | 23 | 0.996 | 0.07 |
|  | United Kingdom | 7 | 6 | 0.952 | 0.026 |
|  | United States | 35 | 25 | 0.968 | 0.071 |
|  | Italy | 36 | 35 | 0.998 | 0.068 |
|  | Europe | 81 | 68 | 0.985 | 0.077 |
|  | Americas | 35 | 25 | 0.968 | 0.071 |
|  | Asia | 83 | 72 | 0.995 | 0.062 |
|  | All | 199 | 161 | 0.994 | 0.071 |
| M | China | 57 | 44 | 0.982 | 0.112 |
|  | China Sichuan | 34 | 26 | 0.979 | 0.108 |
|  | China Others | 23 | 21 | 0.988 | 0.101 |
|  | Belgium | 11 | 3 | 0.345 | 0.015 |
|  | Brazil | 39 | 32 | 0.985 | 0.064 |
|  | Netherlands | 24 | 24 | 1 | 0.065 |
|  | United Kingdom | 23 | 7 | 0.522 | 0.005 |
|  | United States | 36 | 26 | 0.963 | 0.066 |
|  | Americas | 75 | 58 | 0.988 | 0.076 |
|  | Europe | 61 | 34 | 0.886 | 0.062 |
|  | Asia | 61 | 48 | 0.985 | 0.112 |
|  | ALL | 197 | 138 | 0.985 | 0.085 |

Table S9. Population differentiation in FCoV populations sampled from China Sichuan and other regions

| **Gene** | **Region** | **K_ST_** | *P* | **S_nn_** | *P* | **F_ST_** | *P* |
| --- | --- | --- | --- | --- | --- | --- | --- |
| S1 | China mainland | 0.017 | 0.046 * | 0.769 | 0.007 ** | 0.054 | 0.045 * |
|  | China others | 0.030 | 0.015 * | 0.732 | 0.014 * | 0.080 | 0.018 * |
|  | Japan | 0.111 | 0.000 *** | 0.912 | 0.003 ** | 0.344 | 0.000 *** |
|  | Belgium | 0.196 | 0.000 *** | 0.974 | 0.000 *** | 0.336 | 0.000 *** |
|  | Netherlands | 0.053 | 0.000 *** | 0.942 | 0.000 *** | 0.099 | 0.000 *** |
|  | United Kingdom | 0.175 | 0.000 *** | 0.990 | 0.000 *** | 0.399 | 0.000 *** |
|  | Europe | 0.054 | 0.000 *** | 0.932 | 0.000 *** | 0.105 | 0.000 *** |
|  | United States | 0.131 | 0.000 *** | 0.961 | 0.000 *** | 0.243 | 0.000 *** |
| N | China mainland | 0.114 | 0.000 *** | 0.880 | 0.000 *** | 0.198 | 0.000 *** |
|  | China others | 0.101 | 0.000 *** | 0.896 | 0.000 *** | 0.179 | 0.000 *** |
|  | Japan | 0.004 | 0.162 ns | 0.925 | 0.003 ** | 0.121 | 0.000 *** |
|  | Belgium | 0.148 | 0.000 *** | 0.978 | 0.000 *** | 0.508 | 0.000 *** |
|  | Netherlands | 0.041 | 0.000 *** | 0.848 | 0.000 *** | 0.081 | 0.000 *** |
|  | United Kingdom | 0.160 | 0.000 *** | 0.976 | 0.000 *** | 0.394 | 0.000 *** |
|  | Italy | 0.091 | 0.000 *** | 1.000 | 0.000 *** | 0.171 | 0.000 *** |
|  | Europe | 0.039 | 0.000 *** | 0.900 | 0.000 *** | 0.089 | 0.000 *** |
|  | United States | 0.107 | 0.000 *** | 0.913 | 0.000 *** | 0.195 | 0.000 *** |
| M | China mainland | 0.036 | 0.014 * | 0.722 | 0.002 ** | 0.070 | 0.018 * |
|  | China others | 0.033 | 0.018 * | 0.731 | 0.001 ** | 0.063 | 0.009 ** |
|  | Japan | 0.031 | 0.037 * | 0.947 | 0.001 ** | 0.145 | 0.018 * |
|  | Belgium | 0.164 | 0.000 *** | 0.978 | 0.000 *** | 0.314 | 0.000 *** |
|  | Netherlands | 0.081 | 0.000 *** | 0.897 | 0.000 *** | 0.148 | 0.000 *** |
|  | United Kingdom | 0.279 | 0.000 *** | 1.000 | 0.000 *** | 0.436 | 0.000 *** |
|  | Europe | 0.103 | 0.000 *** | 0.945 | 0.000 *** | 0.199 | 0.000 *** |
|  | Brazil | 0.165 | 0.000 *** | 1.000 | 0.000 *** | 0.285 | 0.000 *** |
|  | United States | 0.110 | 0.000 *** | 0.909 | 0.000 *** | 0.196 | 0.000 *** |
|  | Americas | 0.098 | 0.000 *** | 0.960 | 0.000 *** | 0.205 | 0.000 *** |

***, 0.01<P<0.05.**

****, 0.001<P<0.01.**

*****, P<0.001.**

**ns, no significant**

Table S10. Recombination information sheet of feline coronavirus and canine coronavirus by RDP5

| **Gene** | **Recombinant** | **Break point ^A^** | **Minor Parental** | **Major Parental** | ***P-value determined using seven different programs*** | | | | | | |
| --- | --- | --- | --- | --- | --- | --- | --- | --- | --- | --- | --- |
|  |  |  |  |  | **RDP** | **Geneconv** | **BootScan** | **Maxchi** | **Chimarera** | **SiScan** | **3Seq** |
| S1 | CCoV_NC0604 CCoV_GQ477367 CCoV_MW383487 CCoV_MT906865 CCoV_LC190907 CCoV_EF192156 | 1~1071 | TGEV_KX900404 | FCoV_GQ152141  CCoV_LC190906  CCoV_MT166674 | 3.63×10^-2^ | 2.24×10^-12^ | 5.33×10^-4^ | 8.96×10^-18^ | 1.17×10^-7^ | 5.70×10^-36^ | 4.27×10^-56^ |
|  | CCoV_EU856361  CCoV_EU924791  CCoV_HQ450376  CCoV_HQ450377 | 1-1144 | TGEV_JQ700304 | CCoV_MF095854 | 2.57×10-^109^ | NS | 1.64×10^-2^ | 2.28×10^-8^ | NS | 2.48×10^-26^ | 1.29×10^-9^ |
|  | CCoV_EU924790  CCoV_EU856362 | 1-1150 | TGEV_KX900398 | CCoV_MF095854 | 2.72×10^-70^ | 3.01×10^-3^ | NS | 9.44×10^-08^ | 1.48×10^-5^ | 2.92×10^-18^ | 6.89×10^-10^ |
|  | FCoV_KY566211 | 1371~1911 | FCoV_KY292377 | FCoV_KY566209 | NS | 1.20×10^-37^ | 4.41×10^-37^ | 7.76×10^-11^ | 5.08×10^-11^ | 4.24×10^-13^ | 6.48×10^-41^ |
|  | CCoV_NC0521 FCoV_GQ152141 FCoV_AB907624 CCoV_EF192155 CCoV_JF682842 | 641-1221 | CCoV_MT114553 | FCoV_MW316850 | NS | 2.21×10^-12^ | NS | 2.44×10^-13^ | NS | 3.25×10^-19^ | 1.19×10^-15^ |
|  | CCoV_KP981644  (CB/05) CCoV_GU146061 | 391-911 | Unknown （CCoV_GY0609） | CCoV_MF095855 | NS | NS | NS | 2.62×10^-7^ | 8.05×10^-8^ | 1.13×10^-8^ | 1.50×10^-10^ |
|  | FIPV_CD0402 | 1461-2271 | FCoV_MW316830 | FCoV_MW030108 | NS | 3.44×10^-9^ | NS | 1.26×10^-9^ | 4.71×10^-8^ | 6.85×10^-16^ | 4.79×10^-4^ |
|  | FIPV_MY0628 FCoV_MW316849 FCoV_MW316852 FCoV_MW316851 FCoV_MW316848 CCoV_MT114538 CCoV_MT114540——CT114552  CCoV_MT166674 CCoV_MT166675 CCoV_MT166676 | 581-1201 | Unknown （CCoV_MF095848） | FCoV_MW316850 | NS | 9.06×10^-8^ | 3.34×10^-5^ | 1.72×10^-12^ | 9.39×10^-13^ | 7.18×10^-19^ | 9.90×10^-22^ |
|  | FCoV_HQ392469 | 1-817 | FCoV_HQ012371**^B^**  FCoV_HQ392472**^B^** | Unknown （FCoV_FJ938062） | 4.58×10^-49^ | 1.54×10^-9^ | 2.22×10^-10^ | 3.37×10^-13^ | NS | 4.32×10^-42^ | NS |
|  | FCoV_FJ938058 | 550-1269 | FCoV_HQ012372**^B^** FCoV_HQ012369**^B^** | FCoV_FJ938062 | 2.05×10^-45^ | 1.90×10^-7^ | 1.24×10^-8^ | 3.30×10^-9^ | 5.68×10^-6^ | 8.42×10^-15^ | NS |
|  | FCoV_MW316834 | 1203-2288 | FCoV_MW316839 | FCoV_MW316835 | 5.37×10^-47^ | 2.56×10^-8^ | 2.27×10^-4^ | 8.78×10^-8^ | 1.11×10^-4^ | 1.59×10^-12^ | 2.27×10^-6^ |
|  | FCoV_FJ938059 | 1-526 | Unknown （FCoV_FJ938062） | FCoV_FJ938052 | NS | 7.90×10^-16^ | 3.19×10^-17^ | 1.03×10^-13^ | 9.40×10^-3^ | NS | 5.00×10^-36^ |
| N | FIPV_DY0612 | 581-1044 | FIPV_CD0525 | FIPV_CD0615 | NS | 5.91×10^-6^ | NS | 2.12×10^-8^ | 1.98×10^-8^ | 4.91×10^-11^ | 3.23×10^-18^ |
|  | FCoV_HQ392469 | 201-731 | Unknown | FCoV_HQ392472^B^  FCoV_HQ012371^B^ | 1.08×10^-3^ | NS | NS | 8.84×10^-8^ | 1.33×10^-7^ | 8.98×10^-6^ | 3.18×10^-9^ |
|  | FCoV_GU017103 | 381-651 | FCoV_GU017121 | FCoV_GU017104 | 7.67×10^-7^ | 2.85×10^-5^ | 4.71×10^-6^ | 3.65×10^-5^ | 3.23×10^-5^ | 1.50×10^-3^ | 8.31×10^-7^ |
|  | FIPV_DY0523 | 741-1145 | FIPV_DY0528 | FIPV_CD0617 | NS | 8.00×10^-4^ | 7.41×10^-4^ | NS | NS | 3.64×10^-4^ | 2.23×10^-4^ |
| M | FCoV_AB781789 | 341 | FCoV_FJ938056 | CCoV_AB781790 | NS | 2.53×10^-5^ | NS | 1.18×10^-10^ | 6.04×10^-8^ | 5.47×10^-14^ | 1.43×10^-24^ |
|  | FIPV_LS0526 | 271 | CCoV_NC0521 | FIPV _DY0528 | NS | 2.19×10^-6^ | 1.68×10^-6^ | 1.79×10^-10^ | NS | 5.23×10^-18^ | 1.22×10^-23^ |
|  | FCoV_AB086904 | 341 | CCoV_KY063618 | FCoV_FJ917525 | NS | 2.51×10^-9^ | 3.15×10^-11^ | 4.73×10^-10^ | 1.38×10^-12^ | 5.37×10^-17^ | 2.04×10^-22^ |
|  | FIPV_MY0628 | 341 | CCoV_MN078151 | FCoV_FJ938052 | NS | 3.35×10^-8^ | 4.03×10^-10^ | 2.46×10^-10^ | 1.67×10^-11^ | 3.98×10^-16^ | 1.92×10^-21^ |
|  | CCoV_EU924790 | 161，471 | Unknown （FCoV_AB781788） | FCoV_GQ152141CCoV_GQ477367 | 3.45×10^-23^ | 1.58×10^-2^ | NS | 9.84×10^-6^ | NS | 1.96×10^-7^ | 3.70×10^-11^ |
|  | FCoV_AB781788 | 211 | CCoV_AB781790 | FCoV_MF457591 | 1.09×10^-8^ | 3.07×10^-7^ | NS | 2.12×10^-7^ | 3.20×10^-8^ | 6.99×10^-11^ | 2.45×10^-15^ |
|  | FCoV_GQ152141 | 541 | FCoV_FJ938062 | CCoV_HQ450376 | NS | 6.74×10^-7^ | NS | 8.11×10^-8^ | 1.23×10^-7^ | NS | 2.06×10^-16^ |

^A^，the break point in alignment sequence;

^B^，It could also be a recombination sequence;

NS, No significant P-value was recorded for this recombination event using this method.

The sequence marked in red is the sequence in our sample.

Table S11. Positive selection analysis of FCoV-I (A), FCoV-II (B), CCoV-IIa (C), CCoV-IIb (D)


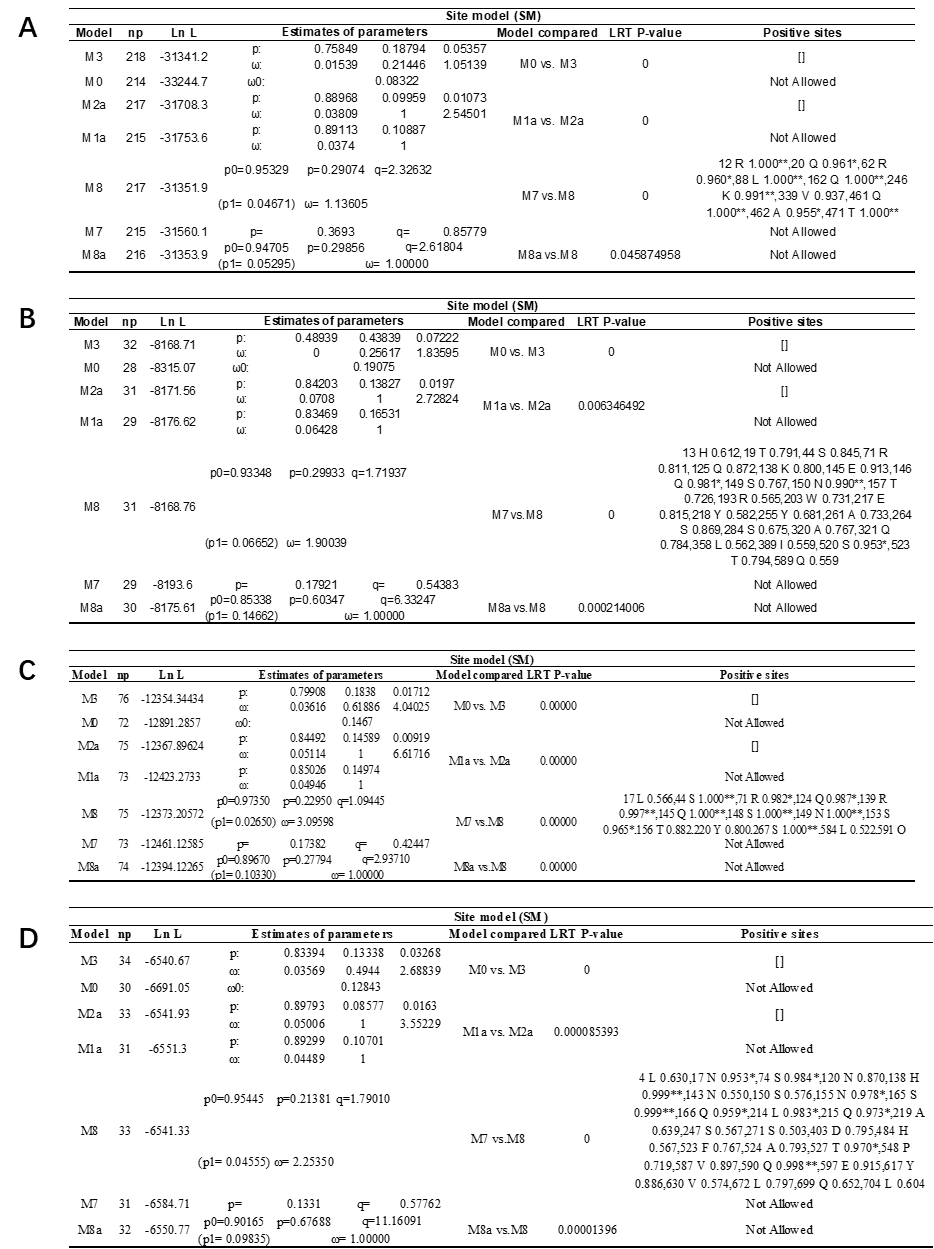


Table S12. Temporal signal testing using Bayesian evaluation of temporal signal (BETs)

| Strain | Model | MLE |
| --- | --- | --- |
| FCoV | Mhet | -38801.06 |
|  | Miso | -41201.35 |
| CCoV | Mhet | -12365.96 |
|  | Miso | -12385.399 |

Table S13 Log marginal likelihoods calculated with path sampling and stepping-stone sampling

| Gene | Colock model | Coalescent model | Path sampling | Stepping-stone sampling |
| --- | --- | --- | --- | --- |
| FCoV-I S1 | Strict | Constant Size | -38801.6564 | -38798.29155 |
|  | Strict | Exponential Growth | -38797.34931 | -38798.68218 |
|  | Strict | Expansion Growth | -38803.0172 | -38797.87203 |
|  | Strict | Bayesian Skyline | -38706.45703 | -38700.05029 |
|  | **Strict** | **Bayesian Skygrid** | **-38648.98949** | **-38662.12745** |
|  | UCLD | Constant Size | -38767.39382 | -38750.66678 |
|  | UCLD | Exponential Growth | -38766.11947 | -38756.58608 |
|  | UCLD | Expansion Growth | -38765.60745 | -38766.77685 |
|  | UCLD | Bayesian Skyline | -38678.31352 | -38677.59121 |
|  | UCLD | Bayesian Skygrid | -38689.81884 | -38687.09004 |
| CCoV-IIa S1 | Strict | Constant Size | -12395.65224 | -12390.38507 |
|  | Strict | Expansion Growth | -12394.83969 | -12391.43927 |
|  | Strict | Exponential Growth | -12397.73889 | -12394.50791 |
|  | Strict | Bayesian Skyline | -12397.24666 | -12393.3419 |
|  | Strict | Bayesian Skygrid | -12393.72831 | -12390.61878 |
|  | UCLD | Constant Size | -12385.03774 | -12381.60113 |
|  | UCLD | Expansion Growth | -12381.52777 | -12376.58191 |
|  | UCLD | Exponential Growth | -12386.1889 | -12380.92396 |
|  | **UCLD** | **Bayesian Skyline** | **-12379.49394** | **-12375.99962** |
|  | UCLD | Bayesian Skygrid | -12381.61769 | -12376.25424 |


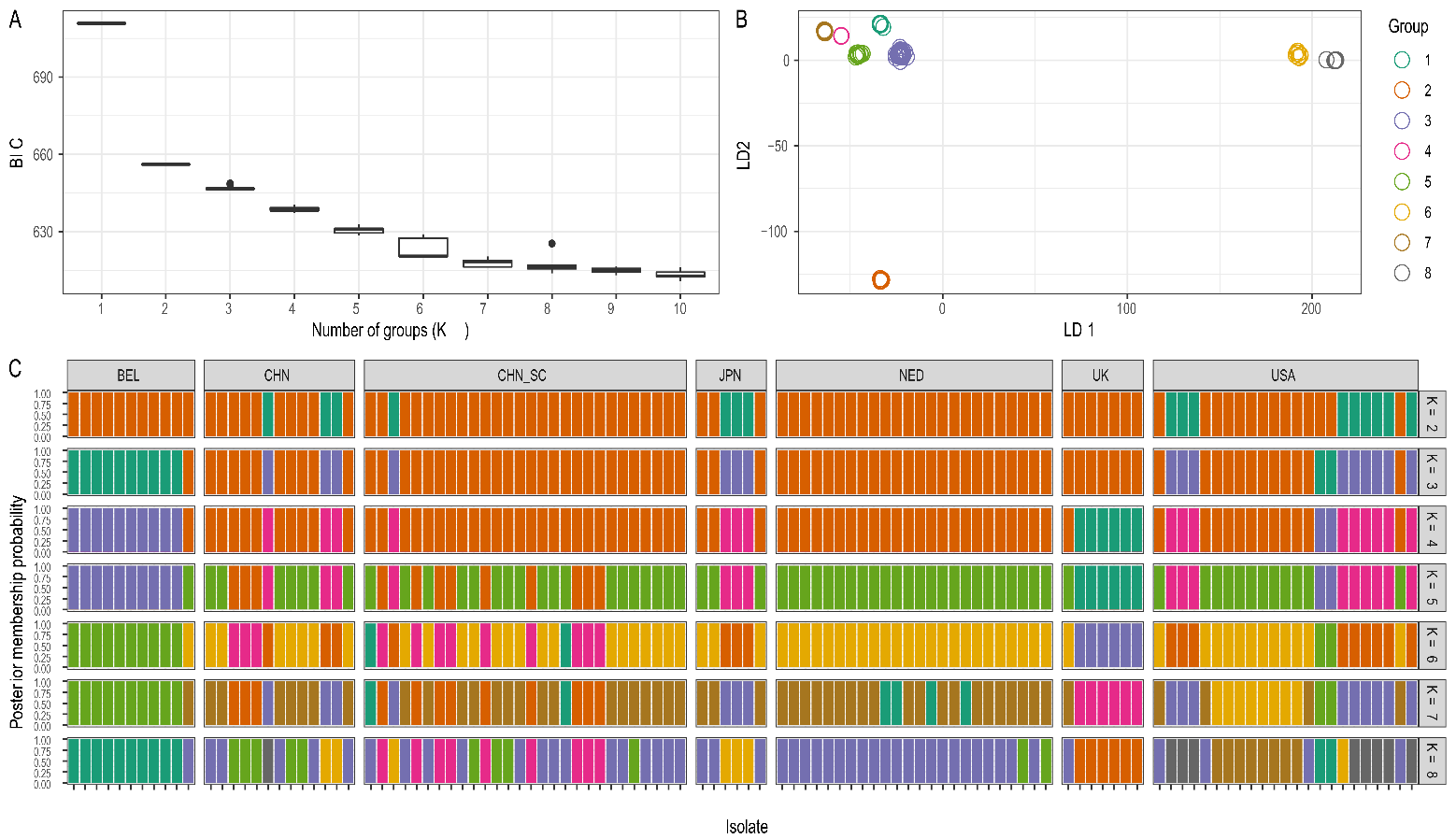


Figure S1. Discriminant analysis of principal components (DAPC) of FCoV S1 gene. All DAPC analysis is done through R package and adegenet library, including K-means cluster analysis, LD analysis to check the clustering situation, and draw the group structure plot. The best cluster K value of FCoV S1 gene is seven.


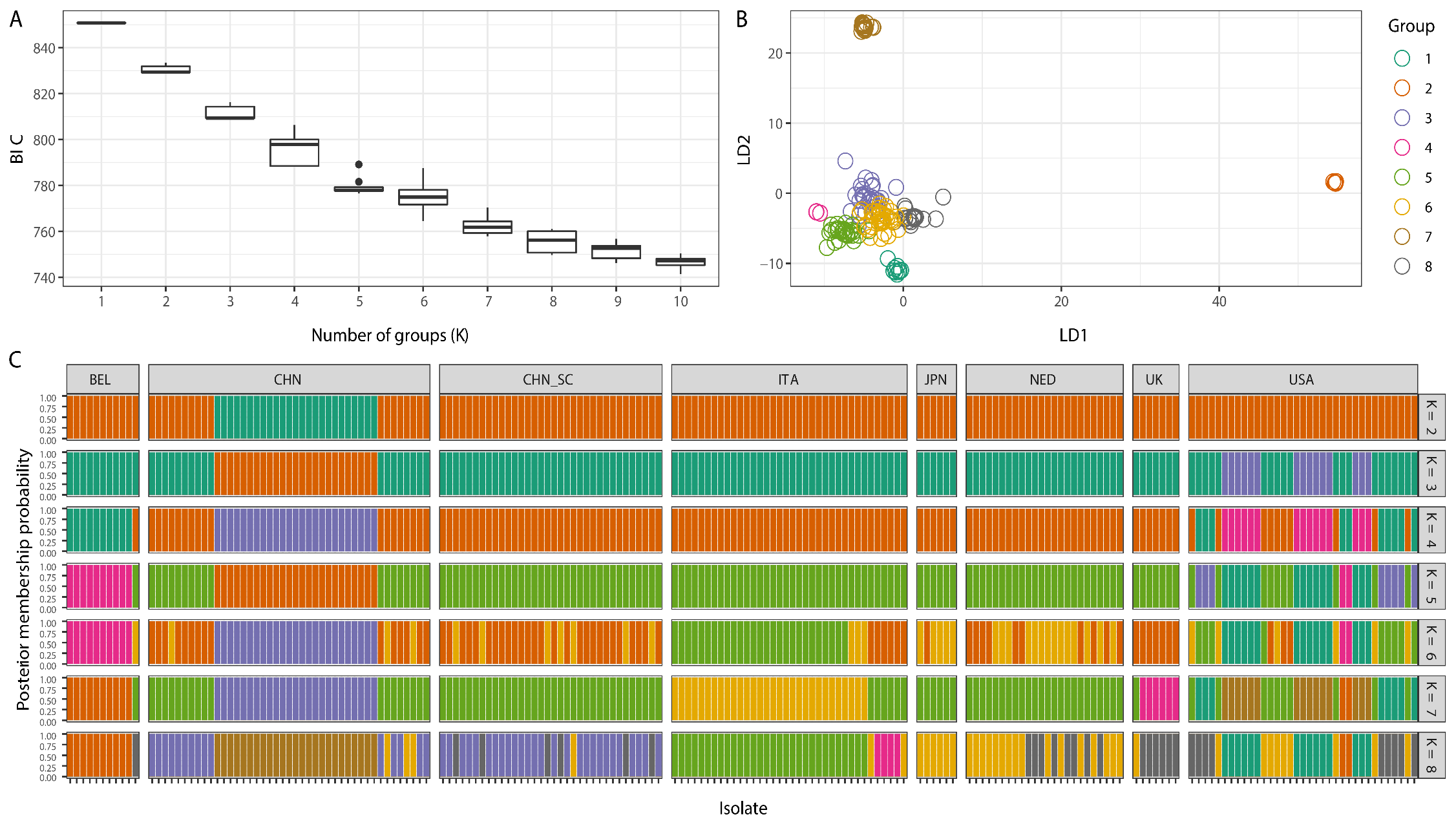


Figure S2. Discriminant analysis of principal components (DAPC) of FCoV N gene. The best cluster K value of FCoV N gene is four.


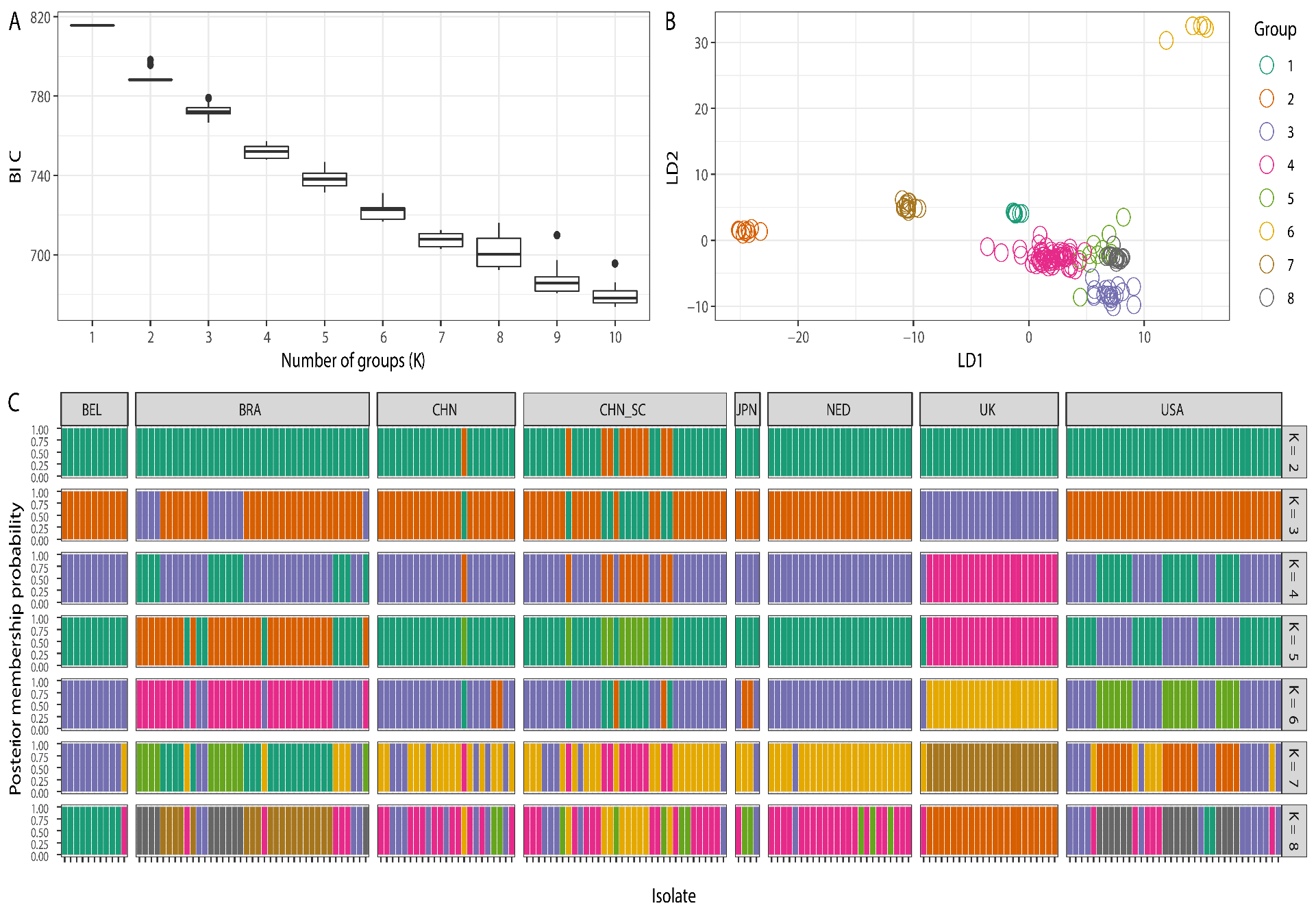


Figure S3. Discriminant analysis of principal components (DAPC) of FCoV M gene. The best cluster K value of FCoV M gene is six.


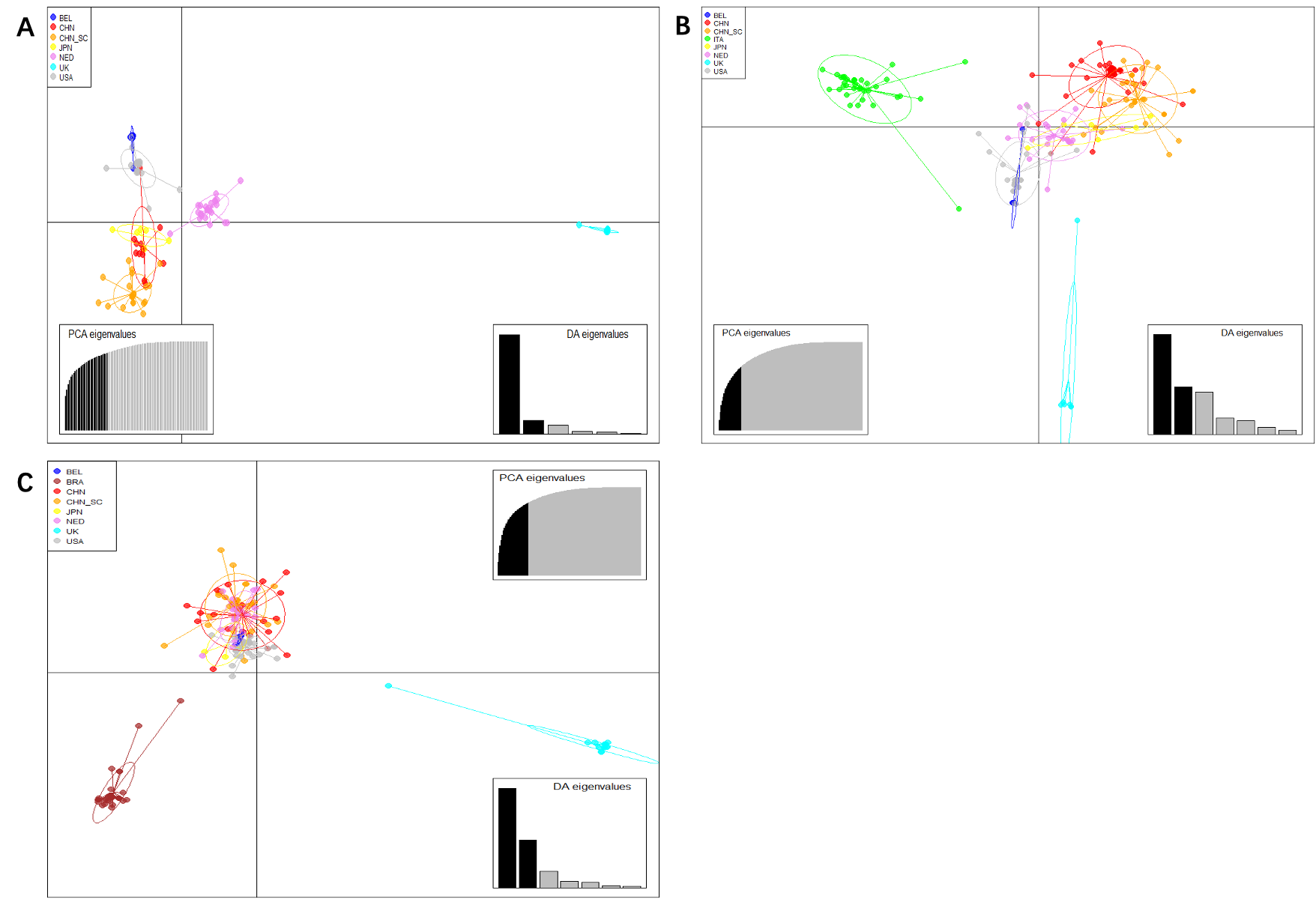


Figure S4. Scatter diagram of the genetic structure of FCoV S1 gene (A), N gene (B) and M gene (C). The scatter plot of genetic structure is also implemented by R package and adegenet library. Individuals (dots) and groups (colours and ellipses) are positioned on the plane using their values for two variables.


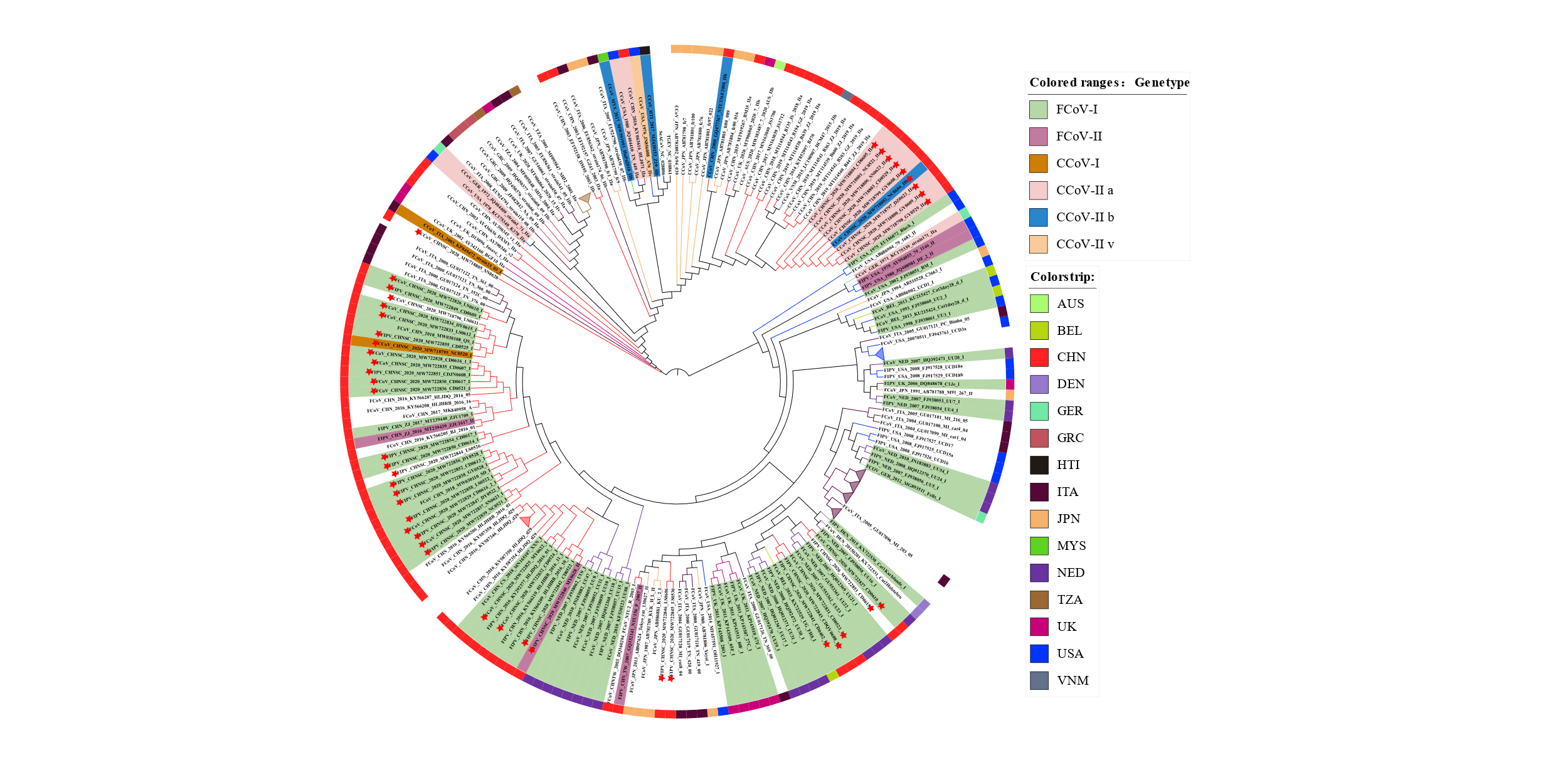

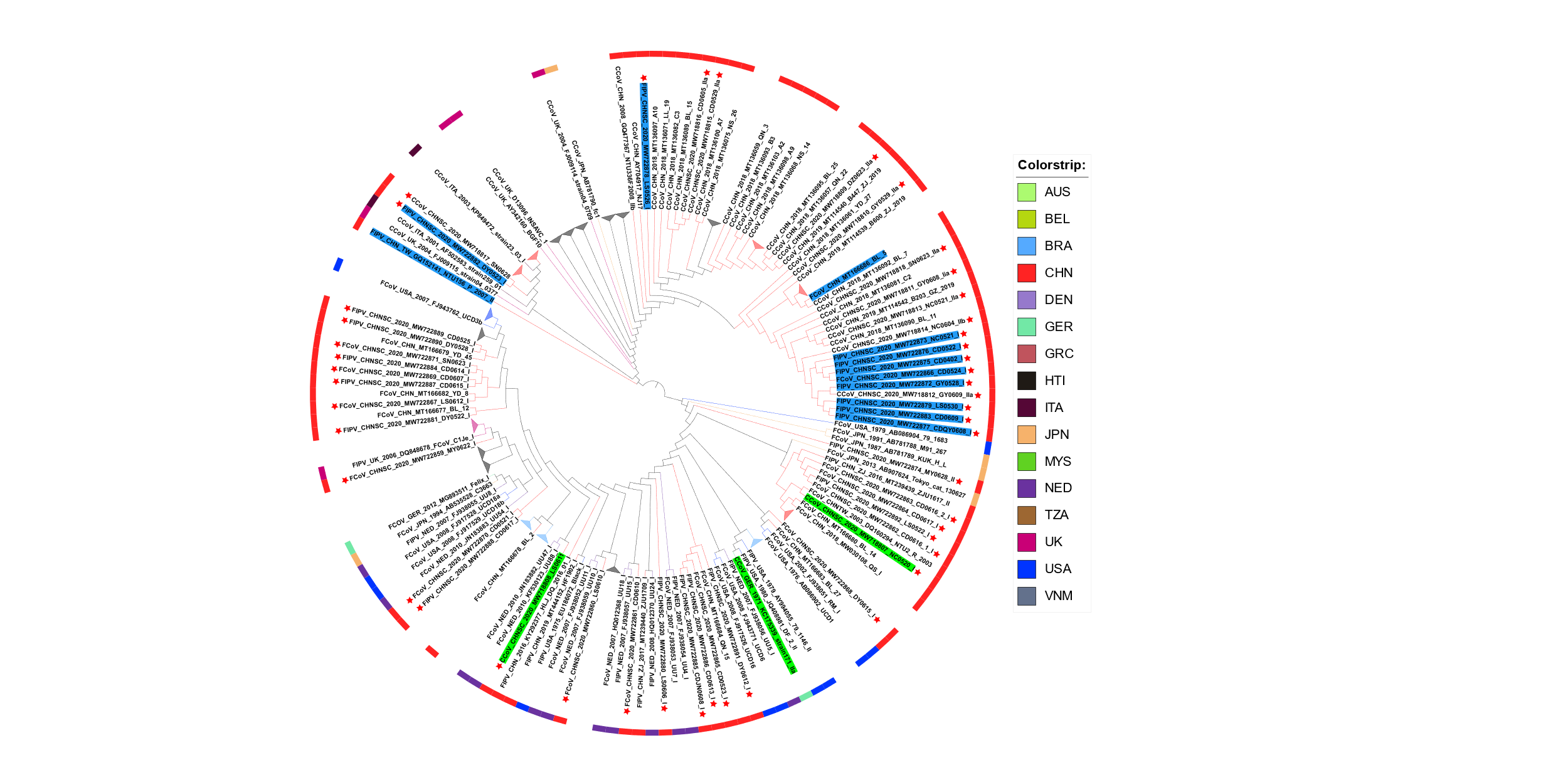


**Figure S5. The maximum likelihood evolutionary tree of FCoV and CCoV N gene.** The ML tree was reconstructed by IQTREE (version 2.1.3) in PhyloSuite (version 1.2.2) with GTR+F+R5 substitution model and 10000 ultrafast bootstrap replicates. Genotypes are distinguished by color range. The color strip and clade color represent the geographic origin of the strain. The red star represents our sample. Each strain is named in the form of species-region-time-accession ID-strain-genotype. Part of the sequence has been folded.

**Figure S6. The maximum likelihood evolutionary tree of FCoV and CCoV M gene.** The ML tree was reconstructed by IQTREE (version 2.1.3) in PhyloSuite (version 1.2.2) with GTR+F+R6 substitution model and 10000 ultrafast bootstrap replicates. The color strip and clade color represent the geographic origin of the strain. The red star represents our sample. Each strain is named in the form of species-region-time-accession ID-strain-genotype. Part of the sequence has been folded.


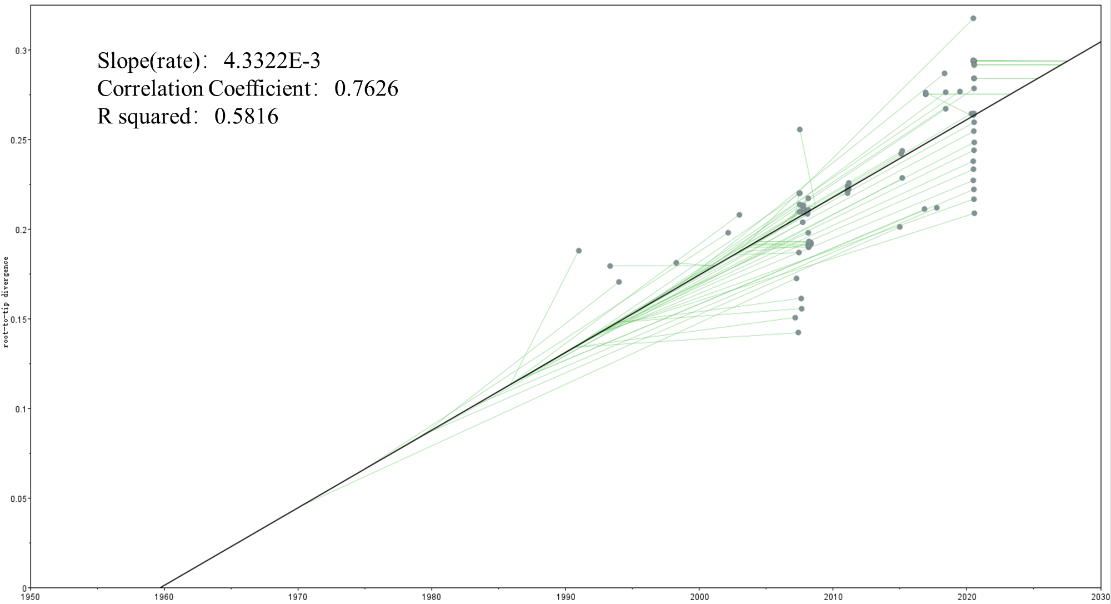

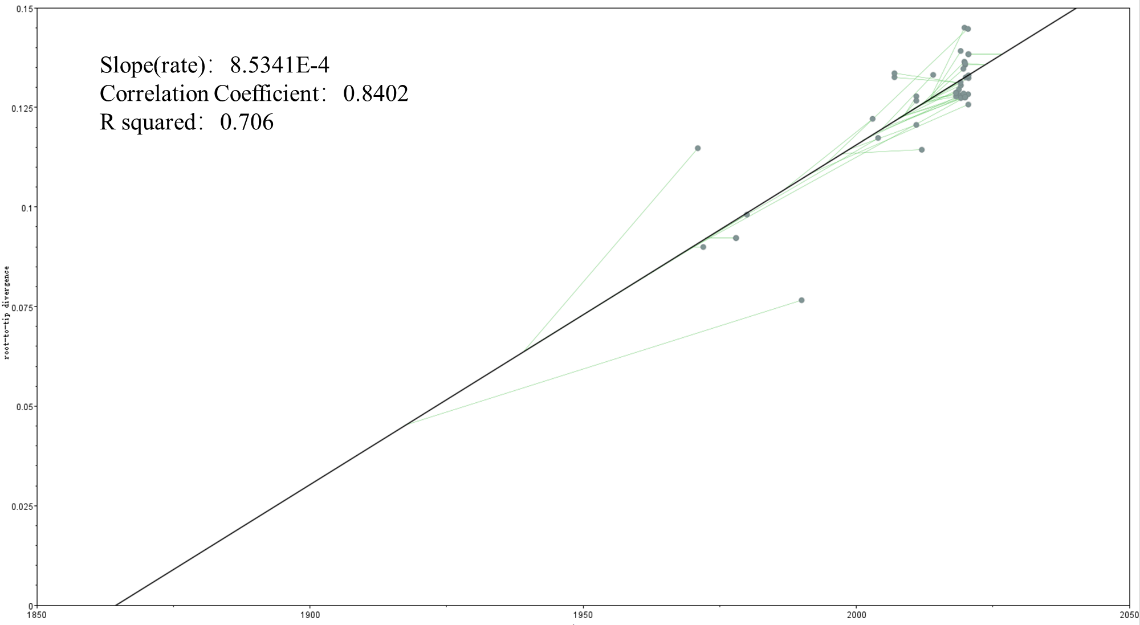


**A**

**B**

**Figure S7. Temporal signal testing based on an ML tree using TempEst (version 1.5.3).** (A) Temporal signal of FCoV-I S1 gene. (B) Temporal signal of CCoV-IIa S1 gene.


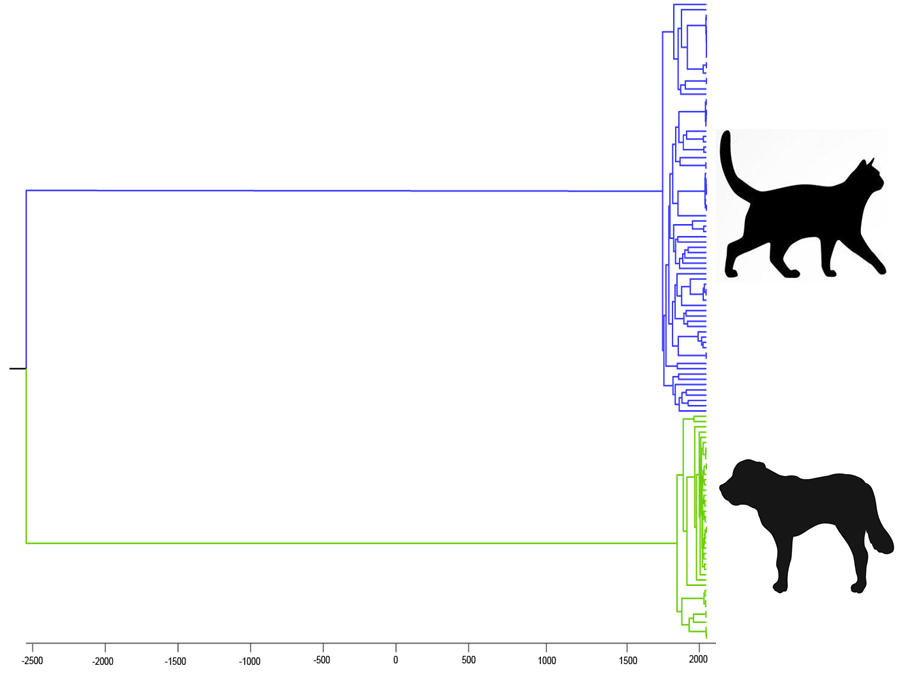


**Figure S8. Maximum clade credibility tree between FCoV-I S1 gene and CCoV-IIa S1 gene.**
